# Revisiting the effects of helper intentions on gratitude and indebtedness: Replication and extensions Registered Report of Tsang (2006)

**DOI:** 10.1098/rsos.250508

**Published:** 2025-04-30

**Authors:** Chi Fung Chan, Hiu Ching Lim, Fung Yee Lau, Wing Ip, Chak Fong Shannon Lui, Katy Yuen Yan Tam, Gilad Feldman

**Affiliations:** ^1^ Department of Psychology, University of Hong Kong, Hong Kong; ^2^ University of Toronto, Toronto, Ontario, Canada; ^3^ Department of Psychology, University of Hong Kong, Hong Kong

**Keywords:** gratitude, indebtedness, intentions, reciprocity, affect, judgement and decision-making, replication, registered report

## Abstract

Gratitude and indebtedness are common emotions in response to a favour, yet research suggests that they are experienced differently depending on the situation. Tsang (Tsang JA. 2006 The effects of helper intention on gratitude and indebtedness. *Motiv. Emot.* **30**, 198–204. (doi:10.1007/s11031-006-9031-z)), found that gratitude for a favour depended on perceived helper intention, whereas indebtedness did not. Perceived benevolent helper intentions were associated with higher gratitude from beneficiaries compared to selfish ones, yet had no associations with indebtedness. In a registered report with a United States Prolific student sample (*n* = 759), we conducted a replication and extensions of studies 2 and 3 from Tsang, 2006. In the original studies, Tsang found support for the impact of the helper’s intention on gratitude (study 2: $\eta_{p}^{2}$ = 0.20 [0.08, 0.32]; study 3: $\eta_{p}^{2}$ = 0.14 [0.03, 0.26]), but not for indebtedness (study 2: $\eta_{p}^{2}$ = 0.01 [0.00, 0.08]; study 3: $\eta_{p}^{2}$ = 0.00 [0.00, 0.03]). In our replications, we found support for the impact of helper’s intention on gratitude (study 2: $\eta_{p}^{2}$ = 0.33 [0.28, 0.37]; study 3: $\eta_{p}^{2}$ = 0.16 [0.12, 0.20]), and—as expected—no support for an effect on indebtedness (study 2: $\eta_{p}^{2}$ = 0.00 [0.00, 0.01]; study 3: $\eta_{p}^{2}$ = 0.01 [0.00, 0.01]). We concluded a successful replication, that helping intent was more strongly associated with gratitude than with indebtedness. Extending the replication, we found evidence for the impact of helper intention on perceived expectations for reciprocity (*d* = 1.51 [1.31, 1.71]), and reciprocity inclination (*d* = 0.66 [0.48, 0.84]), and for opposite associations of perceived reciprocity expectations with gratitude (*r* = −0.28 [−0.35, −0.22]) and indebtedness (*r* = 0.17 [0.10, 0.24]). Materials, data and code are available on: https://osf.io/ghfy4/. This registered report has been officially endorsed by the Peer Community in Registered Reports: https://doi.org/10.24072/pci.rr.100788.

## 1. Background

Gratitude and indebtedness are common emotions in response to receiving help, but studies suggest that they are experienced differently depending on the situation. Tsang [1] showed that helper intentions were associated with feelings of gratitude, yet less so for indebtedness: people reported feeling more grateful when the helper’s intentions were perceived as being less selfish, with weaker to no effects of the helpers’ intentions on feelings of indebtedness.

We conducted a close replication and extension of Tsang [1] with two main goals. Our first goal was to conduct an independent replication of the impact of the helper’s intentions, comparing gratitude and indebtedness. Our second goal was to examine extensions, aiming to enrich our understanding of how differences in helper intentions impact reciprocation.

We begin by introducing the literature on gratitude and indebtedness and the impact of helpers’ intentions on these emotions. We then discuss our motivations for the current replication and review Tsang [1] as our chosen article for replication. Finally, we outline the replication and extension hypotheses, study design and methods.

### 1.1. The effect of helper intention on gratitude and indebtedness

Gratitude and indebtedness are common reactions to receiving help, with these emotions varying across situations [2–4]. Consider, for example, how a student would respond to a classmate who has volunteered to help with homework depends on perceptions of selfish-ulterior intent. The student’s gratitude and indebtedness may depend on whether the act of helping seemed to have been purely benevolent to help another, or rather based on selfish-ulterior intent. These two emotions have often been equated in the early literature and yet evidence showing that these emotions are elicited in different situations suggested the need to differentiate between them [1,5,6].

Gratitude is commonly defined as a positive emotion arising from the appreciation of an action by another person that is desirable and valuable to oneself [7]. McCullough *et al*. [8] argued that it is associated with the prosocial and voluntary nature of the act, as well as the cost incurred and benefits received. Therefore, gratitude may depend on evaluations of the helpers’ costs, altruistic intentions and the value of the favour to the person being helped [9–12]. This is in line with the cognitive perspective that gratitude is defined as the product of the cognition that one has been the beneficiary of others’ goodwill [13].

Greenberg [5] defined indebtedness as a beneficiary’s feeling of obligation to repay the benefactor following norms of reciprocity [14], so as to restore equality in a social exchange [15]. In this context, the favour does not necessarily have to be altruistic. Peng *et al*. [16] suggested that it is the cost of the favour, rather than its intent, that determines the inequality of social exchange, thus affecting feelings of indebtedness.

#### 1.1.1. Relationship and differences between gratitude and indebtedness

Algoe *et al*. [17] posited that gratitude is a positive emotion, whereas indebtedness is a negative emotion. Gratitude leads people to thank their benefactor, whereas indebtedness leads people to try and return the favour. This aligns with the work by Frijda [18] showing that distinct emotions manifest distinct action tendencies—a consequential urge to carry out certain expressive behaviours. It also echoes the research by Gray *et al*. [3] indicating that gratitude is associated with prosocial motivations, whereas indebtedness is associated with avoidance motivations. This was later explained by the broaden-and-build theory by Fredrickson [19], that gratitude, as one of the positive emotions, serves to broaden one’s thoughts and actions to reciprocate, whereas indebtedness is associated with a relatively narrower tit-for-tat reciprocity.

Researchers have tried to distinguish the two emotions in different ways. In terms of their causes, Watkins *et al*. [6] distinguished them by manipulating helpers’ expectations of reciprocity, finding that higher expectations resulted in decreased gratitude yet increased indebtedness. In line with the difference in action tendencies between gratitude and indebtedness, they also found that participants were more likely to express willingness to return the favour if the benefactor communicated weaker reciprocation expectations (reciprocation negatively associated with reciprocation expectations). However, this predicted outcome is arguably counter to combining the theoretical paradigms by Tsang [1] and Algoe *et al*. [17], which together suggest that beneficiaries would be less likely to reciprocate if benefactors held weaker reciprocation expectations (reciprocation positively associated with reciprocation expectations).

Emmons & Crumpler [20] proposed that gratitude is an interpersonal emotion that enhances relational wellbeing, with Mathews & Green [15] arguing that indebtedness is more of a self-focused emotion. Therefore, self-focused people felt less commitment and closeness towards the benefactor. This supported the conclusion by Algoe *et al*. [17] that gratitude enhances relationships, whereas indebtedness only maintains relationships. As shown in the research that distinguishes them, they differ in terms of causes and effects.

Furthermore, there is some research which indicates that these two emotions play different functions in sociality. For example, accumulated literature suggested that gratitude contains a relation-oriented function to promote intimate bonds (e.g. [4,21–24]), whereas indebtedness contains an exchange-oriented function (e.g. [4,25,26]). These functional differences may explain why helpers’ intentions are influential to one’s gratitude and indebtedness.

#### 1.1.2. Perceived helper intention

The differences between gratitude and indebtedness can be reflected in perceived helpers’ intentions. Ames *et al*. [27] found that when beneficiaries perceived helpers’ intention as caring they experienced more positive feelings towards the helper (e.g. happiness and gratitude). Alternatively, perceiving helping intent as manipulative or deceitful triggered negative affect (e.g. indebtedness and anger).

Tsang [1] further examined the effects of helper intentions on feelings of gratitude and indebtedness. The result was partially consistent with Watkins *et al*. [6], with participants feeling more grateful for favours offered with benevolent intentions, compared to favours perceived as having selfish intentions which did not seem to affect indebtedness. However, more recent findings by Welsh *et al*. [28] found helpers’ motives (prosocial versus self-interested) do influence individuals’ levels of indebtedness. They argued that favours with self-interested motives induced less indebtedness than those with prosocial motives, contradicting the work by Tsang [1]. If both findings hold in independent well-powered pre-registered replications, then more work is needed to account for these mixed results.

### 1.2. Choice of study for replication: Tsang (2006)

We embarked on a well-powered close replication and extension registered report of Tsang [1]. We aimed to revisit the phenomenon to examine the reproducibility and replicability of the findings with an independent pre-registered well-powered replication and extension. This follows the recent growing recognition of the importance of reproducibility and replicability in psychological science (e.g. [29,30]).

We chose the study by Tsang [1] based on several factors: its profound academic impact, the absence of direct replications and the realignment in the literature initiated by the article. The article has had an impact on scholarly research in the area of social psychology, and at the time of writing (February 2025), there were 377 Google Scholar citations of the article with many impactful follow-up theoretical and empirical articles. One example is the work by Algoe *et al*. [17] on how gratitude and indebtedness affect romantic relationships. They concluded that gratitude improves interpersonal relationship quality, whereas indebtedness exerts no detectable influence. Based on the findings by Tsang [1], they established a link between the nature of these two emotions and such interpersonal outcomes. That is, the dependence of gratitude on helper’s intentions entails that the focus is on the helper’s positive feelings and favourable mental states (e.g. being generous/caring more for the beneficiary). Meanwhile, indebtedness, being less sensitive to helper’s intentions, focuses on the benefit and thus triggers reciprocity only as a dutiful exchange. Therefore, Tsang [1] contributed to the development of the field by elaborating on the contrast between the two emotions. Her work deepened our understanding of their distinct role in different aspects of life, such as interpersonal relationships. To the best of our knowledge, there are currently no published direct replications of this study.

Despite its impact, the departure of the theory and findings by Tsang [1] from previous research necessitates independent replications to help ensure the realignment is built on solid ground, examines the robustness of the findings and clarifies possible directions for resolution. Prior to Tsang, many studies did not distinguish between the two emotions and instead measured them as one single construct [5,12,31]. The more recent body of research has mostly aligned with the notion that gratitude and indebtedness are distinct emotions.

In addition, the target article presented a theoretical model that predicted no effects for the impact of intent on indebtedness. In their findings, they also reported failing to find a signal in support of rejecting the null hypothesis of finding no differences for indebtedness between the benevolent and selfish intent conditions and built on that to conclude no effects. However, null hypothesis significance testing methods are not well suited for testing and quantifying support for a null hypothesis. We felt it important to revisit the theoretical model by reframing the null hypothesis to differences in effects between gratitude and indebtedness, to rerun the studies with well-powered samples and to add additional analyses that address the null hypothesis issue to gain deeper insights into the phenomenon.

### 1.3. Overview of our replication and extensions

The empirical work by Tsang [1] consisted of three studies, and in the current replication, we focused on studies 2 and 3, which we ran in a single data collection, with the study order randomized to address order effects and to allow us to examine potential consistency between studies. We extended study 3 to test whether helper intentions were associated with reciprocity inclination. In the extension, we proposed and tested five hypotheses, summarized in table 1. We expected to find support for an association between helper intention, gratitude and reciprocation inclination (see table 4 for extension experimental design).

**Table 1.** Summary of effect size and CI of the target article.

Study	Hypothesis	Hypotheses	*p*	Effect size	90% CI	95% CI
2	1a	Gratitude is associated with indebtedness across conditions (selfish-ulterior and benevolent combined)	<0.001*	*r* = 0.57	/	[0.41, 0.69]
	1b	Gratitude is associated with indebtedness in the selfish-ulterior condition	<0.001*	*r* = 0.61	/	[0.39, 0.76]
	1c	Gratitude is [not] associated with indebtedness in benevolent condition. (Reframed from the target article’s null hypothesis)	>0.20	*r* = 0.20	/	[−0.10, 0.47]
	1b + 1c	**Combined: gratitude is more strongly associated with indebtedness in the selfish-ulterior condition than in the benevolent condition**. (Reframed from the target article’s effect/no-effect)		0.61 > 0.20		
	2	Benevolent favours result in more gratitude than selfish-ulterior favours, even after controlling the magnitude of favour	<0.001*	* * $\eta_{p}^{2}=0.2$	[0.08, 0.32]	/
	3	Benevolent favours [do not] result in more indebtedness than selfish-ulterior favours, even after controlling the magnitude of favour. (Reframed from the target article’s null hypothesis)	>0.20	$\eta_{p}^{2}=0.01$	[0.00, 0.08]	/
	**2 + 3**	**Combined: impact of intent on gratitude (benevolent > selfish) is stronger than on indebtedness**. (Reframed from the target article’s effect/no-effect)		0.2 > 0.01		
	4 (2r)	(Regression complementary analysis). Benevolent favours result in more gratitude than selfish-ulterior favours, even after controlling the magnitude of favour	<0.001* (i) <0.01 (ii) <0.01	*R^2^* = 0.73 *β* = 0.32 *β* = 0.62	/	[0.61, 0.81]
	5 (3r)	(Regression complementary analysis). Benevolent favours result in more indebtedness than selfish-ulterior favours, even after controlling the magnitude of favour. (Reframed from the target article’s null hypothesis)	<0.001* (i) >0.20 (ii)<0.001	*R^2^* = 0.26 *β* = 0.13 *β* = 0.42	/	[0.10, 0.41]
	4 + 5 (2r + 3r)	(Regression complementary analysis). The differences between benevolent and selfish-ulterior are stronger for gratitude (H4) than for indebtedness (H5), even after controlling the magnitude of favour		0.32 > 0.13		
3	6	Gratitude is associated with indebtedness in the ambiguous condition	<0.05*	*r* = 0.42	/	[0.06, 0.68]
	7a	Gratitude is different between the three conditions (benevolent, ulterior, and mbiguous)	<0.01*	* $\eta_{p}^{2}=0.14$ *	[0.03, 0.26]	/
	7b	Gratitude is higher in the benevolent condition compared to the ambiguous condition	<0.05*	*d* = 0.55	/	[0.02, 1.08]
	7c	Gratitude is [not] higher in the ambiguous condition compared to the selfish-ulterior condition. (Reframed from the target article’s null result)	0.07	*d* = 0.49	/	[−0.04, 1.01]
	8a	Indebtedness is [not] different between the three conditions (benevolent, selfish, and ambiguous. (Reframed from the target article’s null hypothesis)	>0.20	$\eta_{p}^{2}=0.00$	[0.00, 0.03]	/
	8b	Indebtedness is [not] higher in the benevolent condition compared to the ambiguous condition. (Reframed from the target article’s null hypothesis)	>0.20	*d* = 0.13	/	[−0.39, 0.64]
	8c	Indebtedness is [not] higher in the ambiguous condition compared to the selfish-ulterior condition. (Reframed from the target article’s null hypothesis)	>0.20	*d* = 0.03	/	[−0.49, 0.55]
	**7b/c + 8b/c**	**Combined: impact of intent on gratitude (benevolent > ambiguous > selfish) is stronger than on indebtedness**. (Reframed from the target article’s effect/no-effect)		0.55 > 0.13 |−0.49| > |−0.03|		
	9	Ratings of helper intentions are associated with gratitude in the ambiguous condition	<0.05*	*r* = −0.40	/	[−0.67, −0.04]
	10	Ratings of helper intentions are [not] associated with indebtedness in the ambiguous condition. (Reframed from the target article’s null hypothesis)	>0.20	*r* = 0.00	/	[−0.37, 0.37]
	9 + 10	Combined: Ratings of helper intentions are more strongly associated with gratitude than indebtedness in the ambiguous condition. (Reframed from the target article’s effect/no-effect)		0.40 > 0.00		
Extensions						
3	11a	Competing hypotheses: benevolent helping is perceived as involving *lower* expectations for reciprocation than selfish helping				
	11b	Competing hypotheses: benevolent helping is perceived as involving *higher* expectations for reciprocation than selfish helping				
	12a	Competing hypotheses: benevolent helping leads to *lower* intent to reciprocate than selfish helping				
	12b	Competing hypotheses: benevolent helping leads to *higher* intent to reciprocate than selfish helping				
	13	Lower expectation for reciprocity is associated with a stronger intent to reciprocate				
	14	Higher expectation for reciprocity is associated with less gratitude				
	15	Higher expectation for reciprocity is associated with more indebtedness				

Note: all calculations are corrected to two decimal places if possible. Effect = Cohen’s *d* or partial eta squared. CI = confidence Interval. The asterisk refers to hypotheses supported with *p* < 0.05. Bolded hypotheses are the core hypotheses which will be used to test the replicability of the target article. Hypotheses 4, 5, and 4 + 5, are re-analyses of the hypotheses 2r, 3r, and 2r + 3r. Hypotheses 1c, 3, 8a, 8b, and 8c were originally null hypotheses, yet we reframed those to a testable alternative to the null, with indication of the null hypothesis in brackets (e.g. ‘[not]’). Similarly, the combined hypotheses 1b+1c, 2+3, and 7b/c + 8b/c reframed the null hypotheses from 1c, 3, 8a, 8b, and 8c to a testable hypothesis expecting stronger effects for gratitude compared to indebtedness.

### 1.4. Original hypotheses and findings in the target article

Tsang [1] examined how perceived helper intentions are associated with gratitude and indebtedness experienced by the beneficiary. The core hypothesis was that benevolent (versus selfish) intentions were more strongly associated with gratitude than with indebtedness. We focused our replication on the studies 2 and 3 by Tsang [1], given that study 3 contains all the essential experimental designs of study 1 with an extra condition of ambiguous helper intention for investigation. We briefly outline the studies below.

Study 2 examined the effect of perceived helper intentions on levels of gratitude and indebtedness experienced by the beneficiary in real-life situations. It was conducted using an undergraduate sample at Baylor University, asking participants to recall and write about an experience in which someone offered them a valuable favour, randomly assigning participants to either recall a benevolent or a selfish helper. Study 3 had similar research questions to study 2 yet had different experimental designs. Rather than instructing participants to recall favours to them, it presented participants with a scenario describing benefactors’ helping intent, either benevolent or selfish, randomly assigned. It added an extra condition of ambiguous intent as a control condition, allowing participants to make their own inferences regarding the helper’s intentions.

We provided a summary of the hypotheses and their corresponding findings in table 1 (see the electronic supplementary material, Analysis of the original article subsection for further details). The target article had many hypotheses and many associated analyses, and we therefore pre-registered that our replication criteria will focus on the following. In our replication of study 2, our focus was on the comparison of hypotheses 2 and 3: ‘impact of intent (benevolent > selfish) on gratitude is stronger than on indebtedness’. In our replication of study 3, our focus was on the comparison of hypotheses 7b/c and 8b/c: ‘impact of intent (benevolent>ambiguous > selfish) on gratitude is stronger than on indebtedness’.

Given the two studies, we pre-registered our overall strategy to conclude a successful replication if the findings of the two studies are aligned with a signal in the same direction as the target article by Tsang [1], mixed results if only one of two is supported and failed replication if we fail to find support for both studies.

### 1.5. Exploratory extension: effect of helper intentions on reciprocity expectations and inclination

We aimed to extend study 3 by examining the impact of helper intentions (benevolent versus selfish-ulterior versus ambiguous) on reciprocity using two measures: (i) perceived reciprocity expectations, and (ii) inclination to reciprocate.

We built our extension on the findings by Watkins *et al*. [6], who, like Tsang [1], argued that gratitude and indebtedness are distinct, but went further to argue that expectations for reciprocity would increase indebtedness but decrease gratitude. Tying these findings together with the experimental paradigm of Tsang [1], we aimed to examine the associations between perceived expectations for reciprocity and the inclination to reciprocate. If perceived expectations to reciprocate are *positively* associated with inclination to reciprocate then it would, according to Watkins *et al*. [6], be associated with *increased* indebtedness and *decreased* gratitude. However, if perceived expectations to reciprocate are *negatively* associated with inclination to reciprocate, then it would, according to Watkins *et al*. [6], be associated with *decreased* indebtedness and *increased* gratitude. Therefore, if we were to try and tie the two sets of findings together then the more theory consistent association seems to be that inclination to reciprocate is negatively associated with expectations to reciprocate and therefore higher gratitude than indebtedness.

Findings in the literature about the associations between gratitude and reciprocity have so far been mixed. For example, a seminal study by Bartlett & DeSteno [2] illustrated that gratitude is positively associated with reciprocity, whereas Peng *et al*. [16] failed to replicate Bartlett & DeSteno [2] and did not find any support for links with reciprocity for both gratitude and indebtedness. Therefore, our extension could be thought of as a conceptual replication of the Bartlett & DeSteno [2] and Peng *et al*. [16] directions to try and determine whether reciprocity might play a role, using an empirical design from a different study. To the best of our knowledge, there has been no research examining the impact of helper intention on reciprocation inclination.

In summary, our extension ties and contrasts the predictions by Tsang [1] and Watkins *et al*. [6] and by Bartlett & DeSteno [2] and Peng *et al*. [16] to examine (i) the associations between helper intentions and expectations for reciprocity, and (ii) the relationship between reciprocity inclination, gratitude and indebtedness.

### 1.6. Pre-registration and Open Science

We provided all materials, data and code on: https://osf.io/ghfy4/. This registered report was submitted to *Royal Society Open Science* (RSOS) following peer review and recommendation for stage 2 acceptance at the *Peer Community In* (PCI) *Registered Reports*’ platform. Full details of the peer review and recommendation of the paper at PCI Registered Reports may be found at the links below. After submission to the journal, the article received no additional external peer review but was accepted on the basis of the Editor’s recommendation according to the RSOS PCI Registered Reports’ policy (https://royalsocietypublishing.org/rsos/registered-reports#PCIRR). Stage 1 recommendation and review history: Chen [32]; https://rr.peercommunityin.org/articles/rec?id=373 / https://osf.io/uyfvq/ (our frozen pre-registration version of the entire stage 1 packet: https://osf.io/ka2sv/). Stage 2 recommendation and review history: Chen [33]; https://doi.org/10.24072/pci.rr.100788. All measures, manipulations and exclusions conducted for this investigation are reported, and data collection was completed before conducting the data analyses. The project was part of a large mass replications and extensions project, which received ethics approval from the University of Hong Kong (no. EA210265). This registered report was written based on the registered report template by Feldman [34].

## 2. Method

### 2.1. Power and sensitivity analyses

We first calculated effect sizes and conducted a power analysis based on the effects reported in the target article. Effect size and confidence intervals (CIs) were calculated with R (version: 4.1.2; [35]) with the help of a guide by Jané *et al*. [36], and power analyses were then conducted with a combination of R and GPower (version 3.1.9.6; [37]) for the factors that the authors found support for in the target article (i.e. flagged as significant results). Rounding up to the highest minimum sample size required for both studies, we concluded that the minimum required sample size was 264 participants in total. This calculation was based on the effect size of *d* = 0.55, with power of 0.95, *α* = 0.05 and allocation ratio of 1 : 1. We provide more information regarding these calculations in the electronic supplementary material, Power analysis of the original study effect to assess the required sample for replication subsection. To allow for a comparison, the target article’s study 2 had 92 participants, and study 3 had 86 participants.

Given the likelihood that the original effects are overestimated, we used the suggested Simonsohn [38] rule of thumb, even if meant for other designs, and multiplied the estimated required sample of 264 by 2.5 to result in 660. We then aimed for an even larger sample size of 800.

In stage 1, we reported a sensitivity analysis for a sample of 750, expecting that some participants would not complete the survey, which was close to our final sample of 759 we report below. We found that it allowed for the detection of *d* = 0.24 for independent *t*‐test two conditions contrasts for the study 2 design and *f* = 0.14 for a three conditions ANOVA for the study 3 design and *d* = 0.29 for contrasts between conditions with *n* = 250 (all 95% power, *α* = 5%, one-tail), which are typically considered medium effects in social psychology research [36]. These are much smaller effects than those reported in the target article.

### 2.2. Participants

We recruited United States (US) American students online through Prolific, with a final sample of 759 participants (*M*_age_ = 30.47, s.d. = 11.02; 297 males, 439 females; 18 other; five did not disclose). We note that 907 persons began the survey but 148 did not proceed beyond the consent and verifications and therefore had no data to analyse (explained in ‘Procedure’). We did not pre-register any additional exclusion criteria. We summarized a comparison of the target article sample and the replication samples in table 2.

**Table 2.** Differences and similarities between the target article and our replication.

	Tsang [1]	US Prolific workers
Sample size	Study 2: 92 Study 3: 86	759
Geographical origin	Undergraduates studying at Baylor University	US American Prolific students
Gender	Study 2: 16 males, 76 females Study 3: 13 males, 49 females, 24 did not disclose	297 males, 439 females, 18 others and five did not disclose
Median age (years)	Unreported	27
Average age (years)	Unreported	30.47
S.D. age (years)	Unreported	11.02
Age range (years)	Unreported	18−85
Medium (location)	Study 2: University laboratory cubicles Study 3: Unreported	Online
Compensation	Receiving extra course credit for their participation	Nominal payment
Year	2006 or earlier	2024

We targeted US American students using Prolific’s filters. We restricted the location to the US using ‘standard sample’, we set it to ‘nationality: United States’, ‘country of birth: United States’, ‘student status: yes’, ‘minimum approval rate: 90’, ‘maximum approval rate: 100’, ‘minimum previous submissions: 50’, ‘maximum previous submissions: 10000’ (Prolific’s upper limit), ‘place of most time spent before turning 18: United States’.

### 2.3. Design

We ran the two studies together in a single unified data collection. The display of scenarios and conditions was counterbalanced using the randomizer ‘evenly present’ function in Qualtrics. Scenarios were presented in random order and participants were randomly and evenly assigned to the different conditions. This method was previously tested successfully in many of the replications and extensions conducted by our team (e.g. [39,40]) and is especially powerful in addressing concerns about the target sample (naivety, attentiveness, etc.) when some studies from the same target article replicate successfully whereas others do not, as well as in the potential in drawing inferences about the links between the different studies and consistency in participants’ responding to similar psychology paradigms.

#### 2.3.1. Replication

We summarized the experimental design in tables 3 and 4. The replication of study 2 was a between-subject experiment with two conditions (perceived helper intention: benevolent versus selfish-ulterior condition). The replication of study 3 was a between-subject experiment with three conditions (perceived helper intention: benevolent, ambiguous and selfish-ulterior). We provided all measures in the Qualtrics export in the Open Science Framework (OSF) folder.

**Table 3.** Study 2: replication and extension experimental design.

**Independent variable (IV):** Helper intentions (between-subject)	**Benevolent condition** Asked to recall a situation that someone has done something good for a benevolent reason	**Selfish-ulterior condition** Asked to recall a situation that someone has done something good for a selfish reason
**Dependent variables (DV)**	DV1: **Gratitude** ‘Please choose the number by each adjective to indicate the extent to which you feel each of the following emotions right now in reaction to thinking about the past situation:’ emotion adjectives include *grateful*, *thankful*, and *appreciative*. Scale: 1 = *Would feel very little of this emotion*, 7 = *Would feel a lot of this emotion* (source: [8]) DV2: **Indebtedness** ‘Please choose the number by each adjective to indicate the extent to which you feel each of the following emotions right now in reaction to thinking about the past situation:’ emotion adjectives include *indebted* and *obligated*. Scale: 1 = *Would feel very little of this emotion*, 7 = *Would feel a lot of this emotion* (source: [5]) DV3: **Other emotions** ‘Please choose the number by each adjective to indicate the extent to which you feel each of the following emotions right now in reaction to thinking about the past situation:’ emotion adjectives include *calm*, *pleased*, *resentful*, *upse*t, and *annoyed*. Scale: 1 = *Would feel very little of this emotion*, 7 = *Would feel a lot of this emotion* DV4 (manipulation check): **Perceived helpers’ motivations** ‘Please rate the other person’s motivations on the following scale:’ Scale: 1 = *Very concerned about me*, 7 = *Motivated mostly by selfish reasons* DV5 (extension): **Perceived expectations for reciprocity** ‘Please rate your understanding of the other person’s expectations of you to reciprocate’. Scale: 1 = *No expectations to reciprocate*, 7 = *Very high expectations to reciprocate*	
**Covariate (C)**	**Magnitude of the favour** ‘How big of a favour do you think the other person did for you?’ and ‘how costly (in terms of money, time, effort, etc.) do you think this situation was for the person who did something good for you?’ Scale: 1 = *A very small favour*, 7 = *A very big favour*	
**Comprehension checks (CC)**	What type of helping behaviour are you asked to recall? Whose helping behaviour are you asked to recall?	

Note: DV3 was found in the study materials provided by the author but not reported in the target article. Comprehension check questions were newly designed for this replication and extension study and are not from the target article.

**Table 4.** Study 3: Replication and extension experimental design.

**Independent variable (IV):** Helper intentions (between-subject)	**Benevolent condition** ‘You can tell that your friend is really concerned about you and wants to help you out, so you say yes’	**Selfish-ulterior condition** ‘You know that your friend is really doing you this favour in order to borrow your car next weekend, but you really need those textbooks, so you say yes’	**Ambiguous condition** ‘You really need those textbooks, so you say yes. The next weekend that same friend asks you if they can borrow your car to run some errands’
**Dependent variables (DV)**	DV1: **Gratitude** ‘Please choose the number by each adjective to indicate the extent to which you would feel each of these emotions in the scenario you just read:’ emotion adjectives include: *grateful*, *thankful*, and *appreciative*. Scale: 1 = *Would feel very little of this emotion*, 7 = *Would feel a lot of this emotion* (source: [8]) DV2: **Indebtedness** ‘Please choose the number by each adjective to indicate the extent to which you would feel each of these emotions in the scenario you just read:’ emotion adjectives include: *indebted* and *obligated*. Scale: 1 = *Would feel very little of this emotion*, 7 = *Would feel a lot of this emotion* (source: [5]) DV3: **Other emotions** ‘Please choose the number by each adjective to indicate the extent to which you would feel each of these emotions in the scenario you just read:’ emotion adjectives include *calm*, *pleased*, *resentful*, *upset,* and *annoyed*. Scale: 1 = *Would feel very little of this emotion*, 7 = *Would feel a lot of this emotion* DV4 (manipulation check): **Perceived helpers’ motivations** ‘Please rate the friend’s motivations on the following scale:’ Scale: 1 = *Very concerned about me*, 7 = *Motivated mostly by selfish reasons* DV5: **Loaning experience** ‘Have you ever had a friend loan you money for textbooks?’ Choice: *yes* or *no* DV6 (extension): **Perceived expectations for reciprocity** ‘Please rate your understanding of the other person’s expectations of you to reciprocate’. Scale: 1 = *No expectations to reciprocate*, 7 = *Very high expectations to reciprocate* DV7 (extension): **Reciprocity inclination** ‘To what extent would you have the urge to act in the following ways?’ Items include: (i) *I would feel like helping my friend in return*, (ii) *I would feel like giving my friend a gift in return,* and (iii) *I would feel like doing something for my friend in return*. Scale: 1 = *Slight urge*, 7 = *Very strong urge* (source: [6])		
**Covariate (** ***C*** **)**	**Magnitude of the favour** ‘How much of a favour do you think the friend did by giving money for the textbooks?’ Scale: 1 = *A very small favour*, 7 = *A very big favour*		
**Comprehension checks (CC)**	Specific CC questions include: (Q1) ‘How much money did the friend offer to give to help pay for the textbooks?’ (Q2) ‘What was the favour offered in the scenario?’ (Q3) ‘According to the text: why is your friend offering to help you?’		

Note: DV3 and DV5 were found in the materials provided by the author but not reported in the target article. CC-Q1 was extracted from the target article materials. CC-Q2 and Q3 were newly designed for this replication and extension study.

### 2.4. Procedure

We reached out to the author of the target article and are very grateful for the materials she provided which were very helpful in our reconstruction of the studies.

Participants first indicated their consent, with four questions confirming their eligibility, understanding and agreement with study terms, which they had to answer with a ‘yes’ and the required responses in order to proceed to the study. Three of the four questions also served as attention checks, with a randomized display order of the options (yes, no and not sure)—(i) ‘are you able to pay close attention to the details provided and carefully answer questions that follow?’; (ii) ‘do you understand the study outline and are willing to participate in a survey with brief writing and comprehension checks?’; and (iii) ‘are you a native English speaker born, raised, and currently located in the US?’. Failing any of the three attention questions meant that the participants did not indicate consent and therefore could not embark on the study. These were followed by writing or copy-pasting a statement indicating that they understand and agree to the terms, which participants had to enter correctly in order to proceed, with as many attempts as needed. Upon completion of these steps, participants proceeded to begin the survey.

Following consent and qualification questions, participants completed two studies, a replication of study 2 and a replication of study 3 from the target article, in random order. In contrast to the original article where the two studies were conducted separately, we combined the two studies into a single data collection.

In the replication of study 2, participants recalled an experience in the past year in which they felt that ‘someone else had caused, and was controlling, what was happening in the situation’, and ‘the positive consequences of this other person’s actions were important to you’. Participants in the benevolent condition recalled a situation in which ‘the other person was doing something good for you for unselfish reasons’. Participants in the selfish-ulterior condition recalled someone having done something good for them for selfish reasons.

We used comprehension checks to ensure that participants read and understood the instructions, with multiple choice questions that participants had to answer correctly in order for them to proceed to the task. These questions were as follows: ‘what type of helping behaviour are you asked to recall?’ and ‘whose helping behaviour are you asked to recall?’. Then, participants recalled the described experience and rated their thoughts and emotions that they were feeling in that experience. After that, they proceeded to type the details of the situation in the given box. We then asked about their current emotions in response to the recalled experience with the seven-point gratitude and indebtedness scales used in the target article. They also rated the helpers’ intention and the magnitude of the favour in the experience.

In the replication of study 3, participants were randomly assigned to read one of the three scenarios, namely benevolent, selfish-ulterior and ambiguous. We instructed them to imagine themselves in the scenario. We used the scenarios from the target article, followed by comprehension checks, including questions about what favour was offered in the scenario and why the benefactor offered the favour. The remaining dependent measures, including gratitude and indebtedness scales, favour magnitude scale and helper intention scale, were identical to the items provided in study 2.

Finally, they moved on to the extension. We asked about their inclination to reciprocate. We picked three items from the thought/action readiness items [6] relevant to reciprocation. Justifications were included in the Measures section below.

### 2.5. Manipulations of helper intentions

#### 2.5.1. Study 2

We manipulated the perceived helper intentions using a recall task (table 3). Participants in the benevolent condition were expected to rate the helper’s motivations as less selfish in the manipulation checks.

#### 2.5.2. Study 3

We manipulated the helper intentions in a vignette according to the condition assigned (i.e. benevolent, ambiguous and selfish-ulterior conditions; table 4). Participants were expected to rate the helper as less selfish in the benevolent condition compared to the selfish-ulterior condition in the manipulation checks.

### 2.6. Measures

#### 2.6.1. Replication

##### 2.6.1.1. Emotional responses on gratitude and indebtedness

We adopted the gratitude and indebtedness scales used in the target article. Specifically, the gratitude scale consisted of the emotional adjectives ‘grateful’, ‘thankful’ and ‘appreciative’ (study 2: *α* = 0.97; study 3: *α* = 0.91) and the indebtedness scale consisted of ‘indebted’ and ‘obligated’ (study 2: *α* = 0.72; study 3: *α* = 0.65). Both were on a seven-point Likert scale (1 = *would feel very little of this emotion*; to 7 = *would feel a lot of this emotion*). We took an average for these adjectives to get an overall score of gratitude and indebtedness individually.

We also found five emotions, i.e. ‘calm’, ‘pleased’, ‘resentful’, ‘upset’ and ‘annoyed’, which were on the scale from the target article’s materials provided by the author, but they were not included in the analysis of gratitude and indebtedness. We followed the target article and added them to the data collection.

##### 2.6.1.2. Helper intention (manipulation check)

We adopted the helper intention scale from the target article. Participants rated the helper’s intention in the situation from 1 = *very concerned about me;* to 7 = *motivated mostly by selfish reasons*.

##### 2.6.1.3. Magnitude of the favour

We adopted the favour magnitude scale from the original material. Items were as follows: ‘how big of a favor do you think the other person did for you?’, and ‘how costly (in terms of money, time, effort, etc.) do you think this situation was for the person who did something good for you?’ (*α* = 0.71). Participants rated the magnitude of the favour from 1 = *a very small favour;* to 7 = *a very big favour*.

### 2.6.2. Extensions

#### 2.6.2.1. Perceived expectations for reciprocity

We asked subjects to rate their perceived reciprocity expectations of the benefactor from 1 = *no expectations to reciprocate;* to 7 = *very high expectations to reciprocate*. We note that although the target article did not set off to manipulate expectations, study 3 did vary expectations for reciprocity between the conditions with specific mention of such expectations in the selfish condition. As one reviewer noted, this measure could be considered a manipulation check examining the impact of making that expectation explicit in one of the conditions.

#### 2.6.2.2. Inclination to reciprocate

We picked three items from the thought/action readiness items [6] to measure the inclination to reciprocate (*α* = 0.83): ‘I would feel like helping my friend in return’, ‘I would feel like giving my friend a gift in return’ and ‘I would feel like doing something for my friend in return’.

We chose these items based on their relevance to the reciprocation inclination, excluding items about affect (e.g. ‘I would feel like thinking positive thoughts or happy memories about my friend’) and those irrelevant to reciprocity (e.g. ‘I would feel like ignoring my friend’), focusing on those about actual reciprocation. To better suit our study goal of reciprocity, we slightly modified the items by adding the words ‘in return’ at the end of the sentences (e.g. I would feel like helping my friend *in return*). It was originally a five-point Likert scale about the inclination to have certain thoughts and actions. We changed it to seven-point (1 = *slight urge;^1^* to 7 = *very strong urge*) to align this measure with the other measures in the study so as to not confuse participants in shifting scale ranges. We took an average for these items to get an overall score of reciprocity inclination.

### 2.7. Evaluation criteria for replication findings

There were 16 effect sizes calculated from the target study (see table 1). We compared the replication effects with the corresponding original effects calculated from the target article using the criteria set by LeBel *et al*. [41] (see the electronic supplementary material, Replication evaluation).

### 2.8. Replication closeness evaluation and deviations

We deviated from the target article in a few aspects, summarized in table 5. We evaluated the classification of the replications using the criteria by LeBel *et al*. [42], summarized in table 6 (see also the electronic supplementary material, Replication closeness evaluation). We summarized the replication as a ‘close’ replication.

**Table 5.** Comparison of the target article to our replication.

	Target article	Replication	Reasons for change
Study design	Participants completed the studies with pen and paper in the laboratories	Participants completed the studies with an online survey	To reach more and a wider variety of participants; to conduct the studies with lower cost and higher efficiency
Sample characteristics	Sample size: study 2: 92; study 3: 86 Geographical origin: Undergraduates studying at Baylor University	*n =* 759 Students at the online research platform Prolific	Generalizability of results by including a larger more diverse sample of participants
Procedure	Items on gratitude and indebtedness were not randomized	Items on gratitude and indebtedness were randomized	To reduce the order effect
	Study 2 and study 3 were conducted separately	The replication of study 2 and the replication of study 3 were conducted with the same participants in one setting	Potentially explore consistency in participants’ answers across the two studies (whether an answer in one study is predictive of an answer in the other study) and order/decline effects
		The order of the replications of study 2 and study 3 was randomized	To explore and address potential order effects Allows subsequent separate analysis on participants who took study 2/3 as their first presented study
Procedure	No comprehension check	We added comprehension checks for replication of study 2 and study 3	To ensure that the participants read and understood the instructions and scenarios
Conditions	No change	No change	NA

**Table 6.** Classification of the replication, based on LeBel *et al*. [42].

Design facet	Replication	Details of deviation
Effect/hypothesis	Same	
IV construct	Same	
DV construct	Same	
IV operationalization	Same	
DV operationalization	Same	
Population (e.g. age)	Similar	Target article: the study recruited students from Baylor University in the United States Replication: we targeted students on the online research platform Prolific
IV stimuli	Similar	Target article: two groups of subjects were recruited to receive stimuli from study 2 and study 3, respectively Replication: the same participants answered both the replication of study 2 and the replication of study 3 Target article: items not randomized Replication: items randomized
DV stimuli	Same	
Procedural details	Similar	Target article: one comprehension check Replication: one extra comprehension check was added
Physical settings	Different	Target article: participants completed the studies with pen and paper in a laboratory setting Replication: participants completed the studies online, recruited through Prolific
Contextual variables	Different	Different time and context
**Replication classification**	**Close replication**	

### 2.9. Data analysis strategy

#### 2.9.1. Replication: as in the target article

In both the replication of study 2 and the replication of study 3, to mirror the target article’s analyses, we first ran (Pearson’s) correlation tests to examine the associations between gratitude and indebtedness across conditions and then in the separate benevolent and selfish helper intention conditions.

In study 2, we used ANCOVAs to examine the effect of helper intention (benevolent versus selfish) on gratitude and indebtedness, with the magnitude of favour as the covariate. We supplemented those with regression analyses using the same factors which served a similar purpose to the ANCOVA and merely meant to mirror that target article’s analyses and reported effects.

In study 3, we used one-way ANOVAs to examine the impact of helper intention (benevolent versus selfish-ulterior versus ambiguous) on gratitude and indebtedness. After that, we conducted planned comparisons to examine the differences in emotions between helper intention conditions.

#### 2.9.2. Replication: extension analyses

In both studies in the target article, the comparison between gratitude and indebtedness was done by comparing signals, in which support was found for intent as affecting gratitude but no support for affecting indebtedness. We reframed this to a comparison of the effects of the two dependent variables. To complement the original analyses, we conducted extension analyses of a two-way mixed ANOVA, with helper intent conditions as a between-subject factor (benevolent versus selfish-ulterior in study 2, and benevolent versus selfish-ulterior versus ambiguous in study 3), emotion type as a within-subject factor (gratitude versus indebtedness), and emotion ratings as the dependent variable.

#### 2.9.3. Extensions

We conducted independent samples’ Welch’s *t*-tests (two-tailed) to examine the differences in perceived expectations for reciprocity and reciprocity inclination respectively between the benevolent and selfish-ulterior conditions. Then, we used correlation tests (Pearson’s) to examine the association between perceived reciprocity expectations and reciprocity inclination with the two emotions.

#### 2.9.4. Order effects and outliers and exclusions

Following our stage 1 pre-registration plan, we did not classify any exclusions or outliers.

One deviation from the target article was that all participants completed all scenarios in random order. We consider this to be a stronger design with many advantages, yet one disadvantage is that answers to one scenario may bias participants’ answers to the following scenarios. We pre-registered that if we fail to find support for our hypotheses that we would run exploratory analyses for the failed study by (i) focusing on the participants that completed that study first and examine order as a moderator (*α* = 0.005), and (ii) excluding those who failed the manipulation checks (*α* = 0.001). We concluded a successful replication, and so according to the pre-registration did not plan for additional order analyses, yet to address a request by a reviewer in stage 2 to help readers better understand the possible impact of order on the findings, we conducted an exploratory analysis of the data focusing on the findings when studies were presented first. We provided Rmarkdown code employing a filter that allows the analysis to run on the full high-power sample or on the subset where studies were presented first, included in our OSF. We compared the set of results and concluded the findings to be highly consistent with no major changes.

## 3. Results

We summarized descriptive statistics in table 7 and statistical tests in tables 8 and 9. Our analyses were all performed with R (version: 4.1.2), and we used ggstatsplot [44] and jamovi [45] jmv package for our analyses and figures.

**Table 7.** Studies 2 and 3 replication and extensions: descriptives.

Replication study and factors	Benevolent	Selfish/ulterior	Ambiguous
Replication of Study 2	(*n* = 381)	(*n* = 378)	
Gratitude	6.54 [0.85]	4.05 [1.92]	
Indebtedness	3.91 [1.80]	3.49 [1.84]	
Perceived helper intention	1.91 [1.39]	5.28 [1.46]	
Magnitude of the favour	4.71 [1.44]	3.53 [1.59]	
Perceived expectations for reciprocity (extension)	2.14 [1.71]	4.78 [1.99]	

Replication of Study 3	(*n* = 251)	(*n* = 254)	(*n* = 254)
Gratitude	6.59 [0.79]	5.48 [1.24]	6.14 [1.09]
Indebtedness	5.09 [1.42]	5.09 [1.58]	5.09 [1.43]
Perceived helper intention	1.71 [1.17]	4.81 [1.36]	3.02 [1.59]
Magnitude of the favour	6.27 [0.99]	5.67 [1.32]	6.16 [1.09]
Perceived expectations for reciprocity (extension)	3.65 [1.79]	5.89 [1.18]	4.83 [1.83]
Reciprocity inclination (extension)	6.44 [0.84]	5.76 [1.2]	6.11 [0.98]

Note: mean [s.d.] (condition sample size).

**Table 8.** Replication: Summary of statistical tests and results interpretation.

Hypothesis	Statistical tests	Target article			Replication			Interpretation
		*p*	Effect size	CI	*p*	Effect size	CI	
1a	Pearson correlation	<0.001*	*r* = 0.57	[0.41, 0.69]	<0.001	*r =* 0.34	[0.28, 0.40]	Signal—inconsistent, smaller
1b	Pearson correlation	<0.001*	*r* = 0.61	[0.39, 0.76]	<0.001	*r* = 0.53	[0.46, 0.60]	Signal—inconsistent, smaller
1c	Pearson correlation	>0.20	*r* = 0.20	[−0.10, 0.47]	= 0.70	*r* = 0.02	[−0.08, 0.12]	No signal—consistent
2	ANCOVA	<0.001*	$\eta_{p}^{2}=0.2$	[0.08, 0.32]	<0.001	* $\eta_{p}^{2}=0.33$*	[0.28, 0.37]	Signal—inconsistent, larger
3	ANCOVA	>0.20	* $\eta_{p}^{2}=0.01$*	[0.00, 0.08]	= 0.54	$\eta_{p}^{2}\;<0.001$	[0.00, 0.01]	No signal—consistent
2 + 3	Mixed ANOVA (extension)	/	*/*	/	<0.001	* $\eta_{p}^{2}=0.23$*	[0.19, 0.27]	**Fully supported**
4	Linear regression	<0.0001* (i) <0.01 (ii) <0.01	*R^2^* = 0.73 *β* = 0.32 *β* = 0.62	[0.61, 0.81]	<0.001 (i) <0.001 (ii) <0.001	*R^2^* = 0.53 β = −0.51 β = 0.37	[0.48, 0.58]	Signal—inconsistent, smaller
5	Linear regression	<0.001* (i) >0.20 (ii) <0.001	*R^2^* = 0.26 *β* = 0.13 *β* = 0.42	[0.10, 0.41]	<0.0001 (i) = 0.540 (ii) <0.001	*R^2^* = 0.14 β = 0.02 β = 0.38	[0.10, 0.19]	Signal—inconsistent, smaller
6	Pearson correlation	<0.05*	*r* = 0.42	[0.06, 0.68]	<0.001	*r* = 0.23	[0.11, 0.34]	Signal—inconsistent, smaller
7a	One-way ANOVA	<0.01*	* $\eta_{p}^{2}=0.14$*	[0.03, 0.26]	<0.001	* $\eta_{p}^{2}=0.16$*	[0.12, 0.20]	Signal—consistent
7b	Independent *t*‐test (two-tailed)	<0.05*	*d* = 0.55	[0.02, 1.08]	<0.001	*d* = 0.42	[0.25, 0.60]	Signal—consistent
7c	Independent *t*‐test (two-tailed)	0.07	*d* = 0.49	[−0.04, 1.01]	<0.001	*d* = 0.63	[0.45, 0.81]	Signal—inconsistent, positive effect
7b/c + 8b/c	Mixed ANOVA (extension)	/	/	/	<0.001	$\eta_{p}^{2}=\;0.07$	[0.05, 0.10]	**Fully supported**
8a	One-way ANOVA	>0.20	$\eta_{p}^{2}=0.00$	[0.00, 0.03]	= 1.00	* $\eta_{p}^{2}<0.01$*	[0.00, 1.00]	No signal—consistent
8b	Independent *t*‐test (two-tailed)	>0.20	*d* = 0.13	[−0.39, 0.64]	= 0.994	*d* = 0.00	[−0.17, 0.18]	No signal—consistent
8c	Independent *t*‐test (two-tailed)	>0.20	*d* = 0.03	[−0.49, 0.55]	= 0.969	*d* = 0.00	[−0.18, 0.16]	No signal—consistent
9	Pearson correlation	<0.05*	*r* = −0.40	[−0.67, −0.04]	<0.001	*r* = −0.50	[−0.59, −0.40]	Signal—consistent
10	Pearson correlation	>0.20	*r* = 0.00	[−0.37, 0.37]	= 0.80	*r =* −0.21	[−0.14, 0.11]	No signal—consistent

Note: see table 1 for hypotheses. For partial eta-squared, we report 90% CIs instead of 95% in order not to include zero with the *p*-value falling below 0.05 [43] Lakens, 2014. The interpretation of replication outcome is based on an evaluation criteria by LeBel *et al*. [41].

**Table 9.** Extensions: summary of statistical tests.

Hypothesis	Stat. tests	d.f.	*p*	Effect size	CI
11	Independent *t*‐test (two-tailed)	433	<0.001	*d* = 1.51	[1.31, 1.71]
12	Independent *t*‐test (two-tailed)	455	<0.001	*d* = 0.66	[0.48, 0.84]
13	Pearson correlation	757	=0.08	*r =* −0.06	[−0.13, 0.01]
14	Pearson correlation	757	<0.001	*r =* −0.28	[−0.35, −0.22]
15	Pearson correlation	757	<0.001	*r =* 0.17	[0.10, 0.24]

Note: see table 1 for all hypotheses. CI = 95% confidence intervals.

### 3.1. Replication

#### 3.1.1. Study 2

##### 3.1.1.1. Manipulation check: helper intention

We conducted an independent samples *t*‐test (Welch’s*,* two-tailed) and found that participants in the benevolent condition rated the helper’s motivations as less selfish (*n* = 381, *M* = 1.91, s.d. = 1.39) than in the selfish-ulterior condition (*n* = 378, *M* = 5.28, s.d. = 1.46; *M_d_* = −3.37; *t*_754_ = −33, *p* < 0.001; *d* = −2.40, 95% CI [−2.21, −2.58]).

We also found that participants rated the magnitude of favour as larger in the benevolent condition (*n* = 381; *M* = 4.71, s.d. = 1.44) than in the selfish-ulterior condition (*n* = 378; *M* = 3.53, s.d. = 1.59; *M_d_* = 1.18; *t*_748_ = 11, *p* < 0.001; *d* = 0.80, 95% CI [0.65, 0.95]).

##### 3.1.1.2. Associations between gratitude and indebtedness

**Figure 1.**
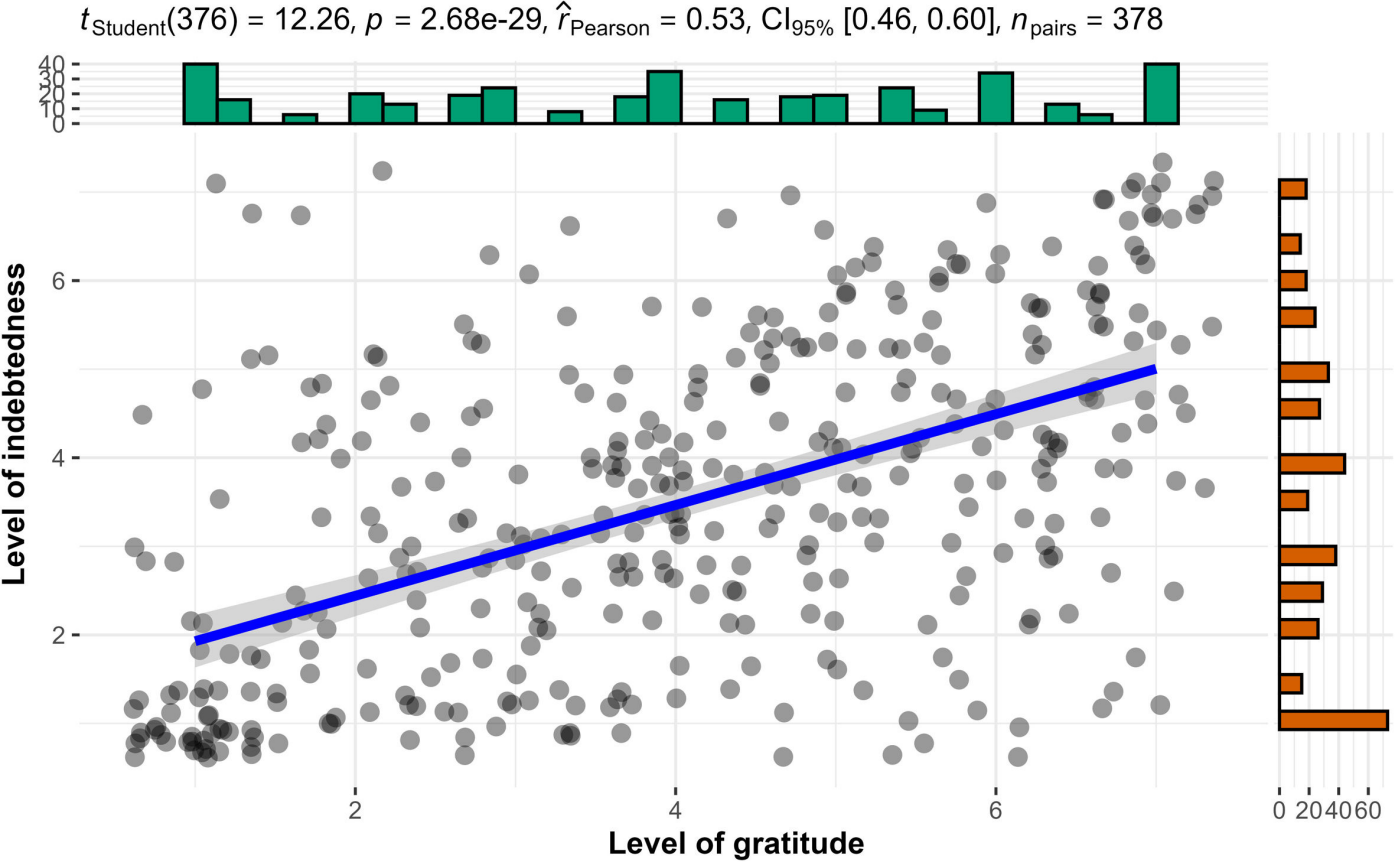
Study 2 selfish-ulterior condition (H1b): association between gratitude and indebtedness.

**Figure 2.**
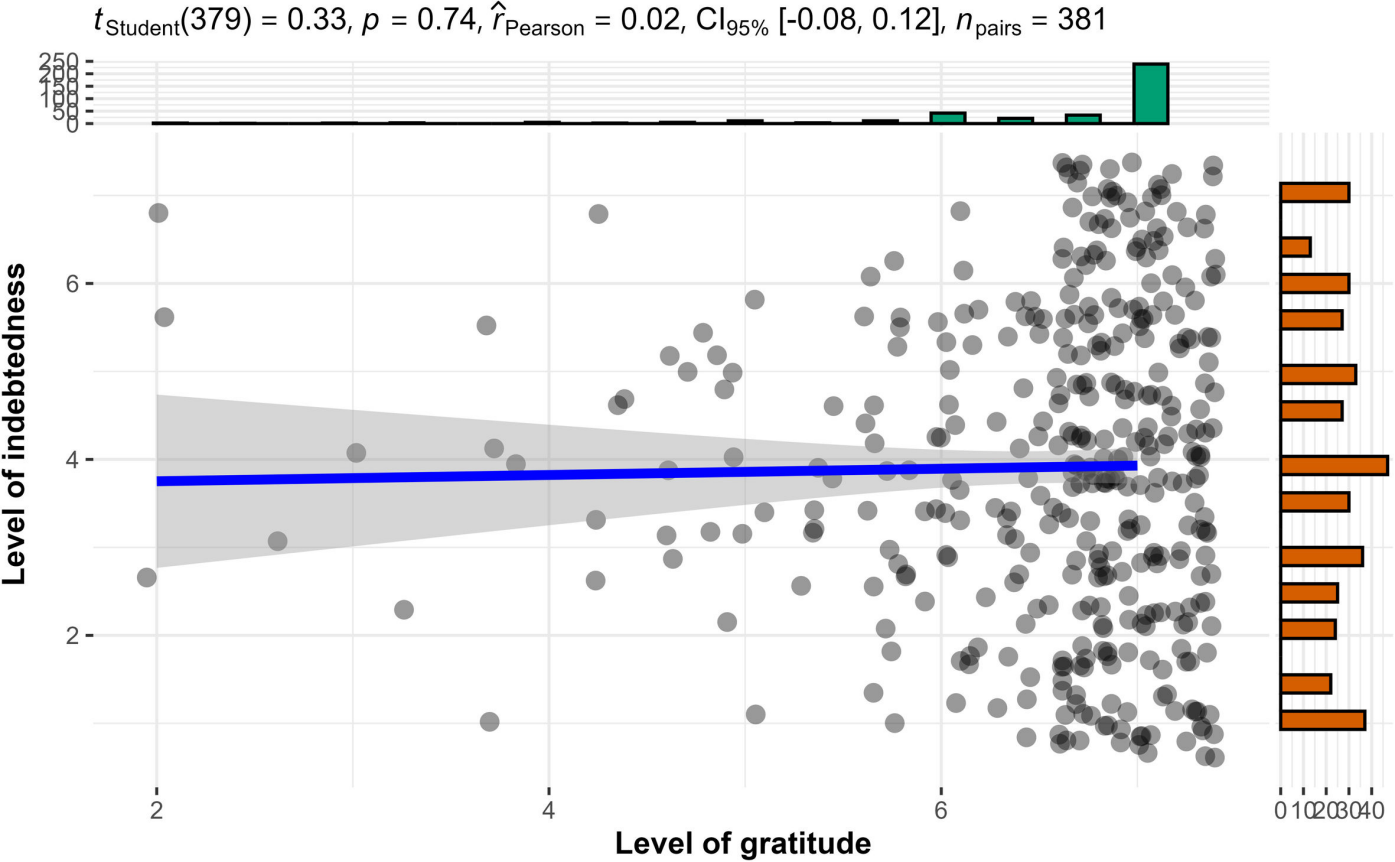
Study 2 benevolent condition (H1c null hypothesis): association between gratitude and indebtedness.

We conducted Pearson’s correlation tests and found support for a positive association between gratitude and indebtedness, both across conditions (hypothesis 1a: *r*_757_ = 0.34, 95% CI [0.28, 0.40], *p* < 0.001), and in the selfish-ulterior condition (hypothesis 1b: *r*_376_ = 0.53, 95% CI [0.46, 0.60], *p* < 0.001; figure 1), and as expected we failed to find support for an association in the benevolent condition (hypothesis 1c reframed from a null hypothesis: *r*_379_ = 0.02, 95% CI [−0.08, 0.12], *p* = 0.7; figure 2).

##### 3.1.1.3. Core hypothesis: impact of helper intent (benevolent > selfish) on gratitude is stronger than on indebtedness

**Figure 3.**
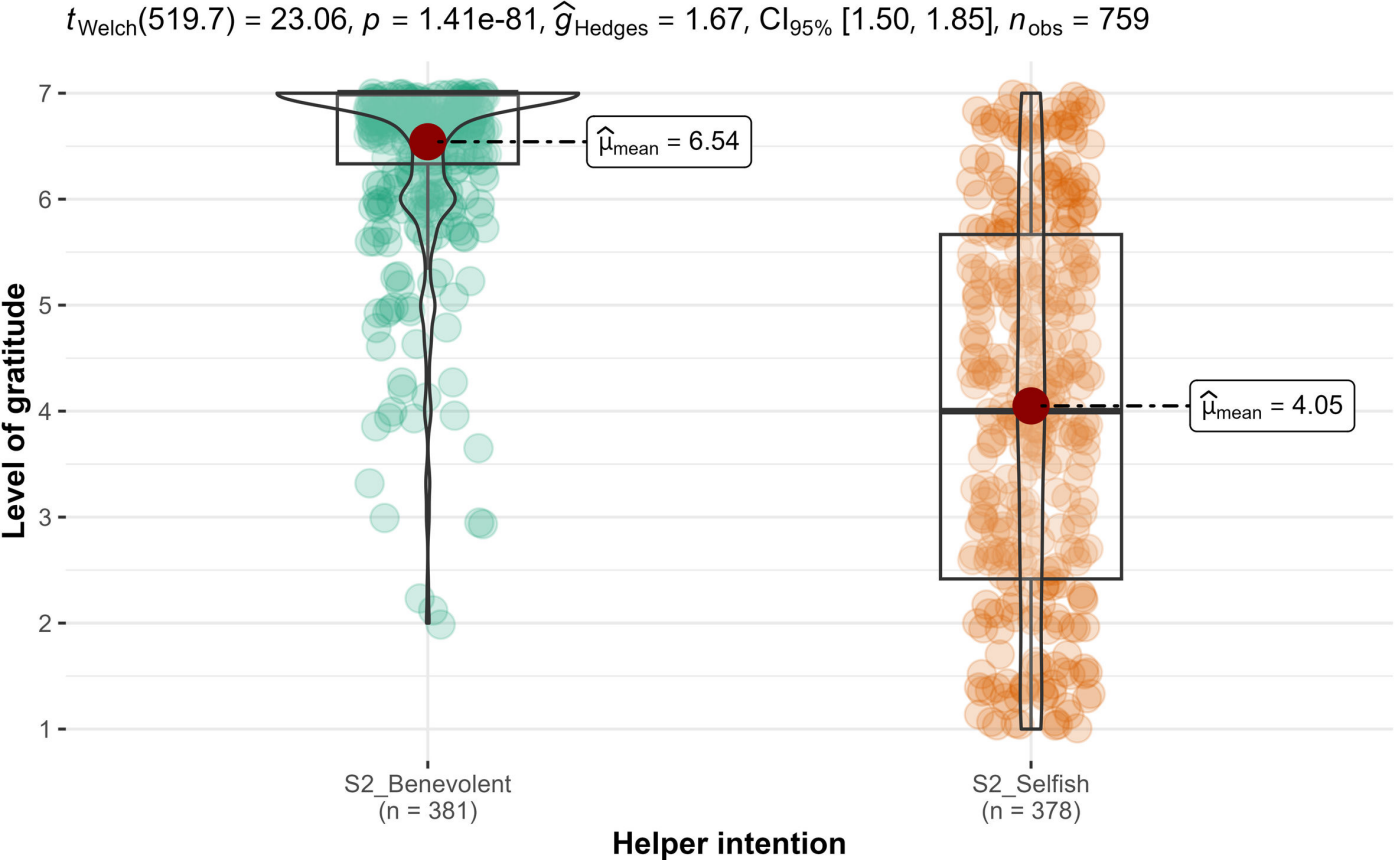
Study 2: helper intention impact on gratitude. Note: scale is from 1 to 7; higher values indicate stronger feelings of gratitude.

**Figure 4.**
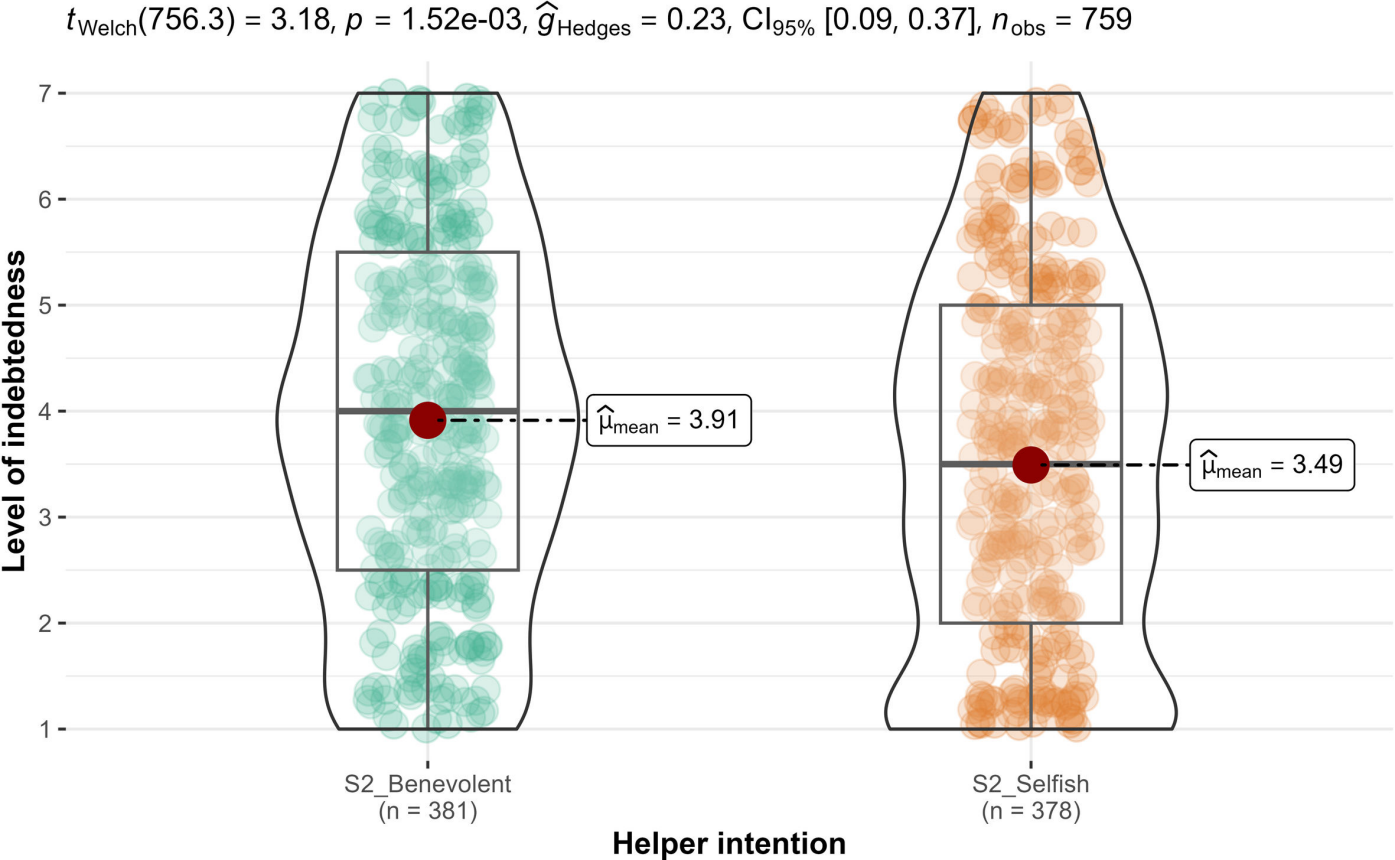
Study 2: helper intention impact on indebtedness. Note: scale is from 1 to 7; higher values indicate stronger feelings of indebtedness.

We conducted ANCOVAs, with the rated magnitude of favour as a covariate and found support for differences in gratitude between the benevolent condition and the selfish-ulterior condition (H2: *F*_1, 756_ = 365, *p* < 0.001; $\eta_{p}^{2}$* *= 0.33, 90% CI [0.28, 0.37]; figure 3), but not in indebtedness (H3 null hypothesis: *F*_1, 756_ = 0.37, *p* = 0.54; $\eta_{p}^{2}$* *= 0.001, 90% CI [0.00, 0.01]; figure 4). We, therefore, concluded support for the combination of hypotheses 2 and 3 for higher gratitude when recalling a benevolent favour compared to a selfish favour, after controlling for the magnitude of the favour, but less so for indebtedness.

##### 3.1.1.4. Complementary regression analyses

We conducted regression analyses with a condition variable contrasting benevolent and selfish helper intent and magnitude of favour predicting gratitude and indebtedness. For gratitude, we found support for both intention conditions (*β* = −0.51, *t*_756_ = −19.11, *p* < 0.001) and magnitude of favour (*β* = 0.37, *t*_756_ = 13.84, *p* < 0.001) as predictors of feelings of gratitude (*R^2^* = 0.53, 95% CI [0.48, 0.58], *F*_2, 756_ = 430.49, *p* < 0.001).

We found support for magnitude of favour (*β* = 0.38, *t*_756_ = 10.46, *p* < 0.001), but not for intention conditions (*β* = 0.02, *t*_756_ = 0.61, *p* = 0.54), as predictors of feelings of indebtedness (*R^2^* = 0.14, 95% CI [0.10, 0.19], *F*_2, 756_ = 60.52, *p* < 0.001).

We concluded support for hypothesis 4 (2r), that helpers’ intent (benevolent versus selfish-ulterior) and magnitude of favour predict gratitude, but—as expected—not for hypothesis 5 (3r null hypothesis) that helpers’ intent predicts indebtedness.

##### 3.1.1.5. Interaction between intent and emotions (gratitude versus indebtedness): extension analysis of a direct test to core hypothesis

**Figure 5.**
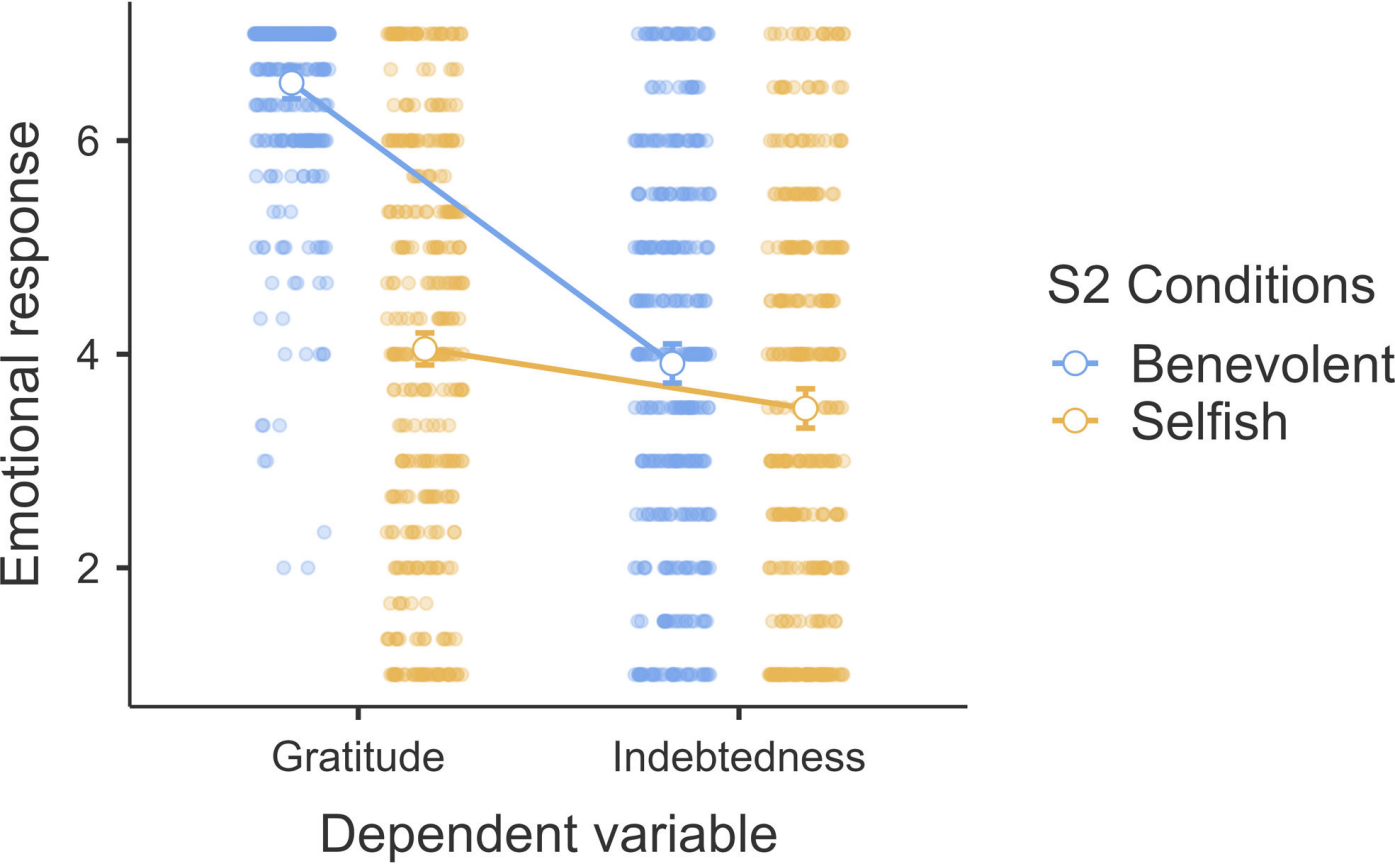
Study 2: interaction between helper intention and emotions. Note: scale is from 1 to 7; higher values indicate stronger feelings of the emotion.

We conducted a mixed ANOVA examining the interaction between intent (benevolent versus selfish-ulterior; between-subject) and emotion type (gratitude versus indebtedness; repeated) and found evidence of main effect for emotion type (*F*_1, 757_ = 532.4, *p* < 0.001, $\eta_{p}^{2}$ = 0.41, 95% CI [0.37, 0.46]), evidence of main effect for intent (*F*_1, 757_ = 216.6, *p* < 0.001, $\eta_{p}^{2}$* *= 0.22, 95% CI [0.18, 0.27]) and evidence of interaction between intent and emotion type (*F*_1, 757_ = 225.0, *p* < 0.001, $\eta_{p}^{2}$* *= 0.23, 95% CI [0.19, 0.27]). We plotted the findings in figure 5.

### 3.1.2. Study 3

#### 3.1.2.1. Manipulation check: helper intention

We conducted independent samples *t*-tests (Welch’s; two-tailed) and found that participants rated benefactor as being less selfish in the benevolent condition (*n* = 251; *M* = 1.71, s.d. = 1.17) compared to the selfish-ulterior condition (*n* = 254; *M* = 4.81, s.d. = 1.36; *M_d_* = 3.10; *t*_493_ = 28, *p* < 0.001; *d* = 2.49, 95% CI [2.26, 2.72]). We also found that participants rated helper as being more selfish in the selfish-ulterior condition compared to the ambiguous condition (*n* = 254; *M* = 3.02, s.d. = 1.59; *M_d_* = 1.79; *t*_494_ = 14, *p* < 0.001; *d* = 1.24, 95% CI [1.05, 1.43]). Participants rated helpers as being less selfish in the benevolent condition compared to the ambiguous condition (*t*_464_ = −11, *p* < 0.001; *d* = −0.98, 95% CI [−1.16, −0.79]).

#### 3.1.2.2. Covariate: magnitude of favour

We conducted independent samples *t*-tests (Welch’s; two-tailed) and found that the rated magnitude of favour in the selfish-ulterior condition (*n* = 254; *M* = 5.67, s.d. = 1.32) was lower than in the benevolent condition (*n* = 251; *M* = 6.27, s.d. = 0.99; *M_d_* = −0.60; *t*_469_ = −5.8, *p* < 0.001; *d* = −0.52, 95% CI [−0.69, −0.34]) and in the ambiguous condition (*n* = 254; *M* = 6.16, s.d. = 1.09; *M_d_* = −0.49; *t*_489_ = −4.5, *p* < 0.001; *d* = −0.40, 95% CI [−0.58, −0.22]). However, we found no support for the magnitude of favour in the benevolent condition as different from that in the ambiguous condition (*t*_499_ = 1.2, *p* = 0.20; *d* = 0.11, 95% CI [−0.07, 0.28]).

#### 3.1.2.3. Associations between gratitude and indebtedness

We conducted Pearson’s correlation tests and found support for a positive association between gratitude and indebtedness in the benevolent condition (*r*_249_ = 0.25, 95% CI [0.13, 0.36], *p* < 0.001), the selfish-ulterior condition (*r*_252_ = 0.17, 95% CI [0.05, 0.29], *p* < 0.01) and the ambiguous condition (*r*_252_ = 0.23, 95% CI [0.11, 0.34], *p* < 0.001). We, therefore, concluded support for hypothesis 6, that gratitude is positively associated with indebtedness in ambiguous conditions.

#### 3.1.2.4. Core hypothesis: impact of intent (benevolent > ambiguous > selfish) on gratitude is stronger than on indebtedness

**Figure 6.**
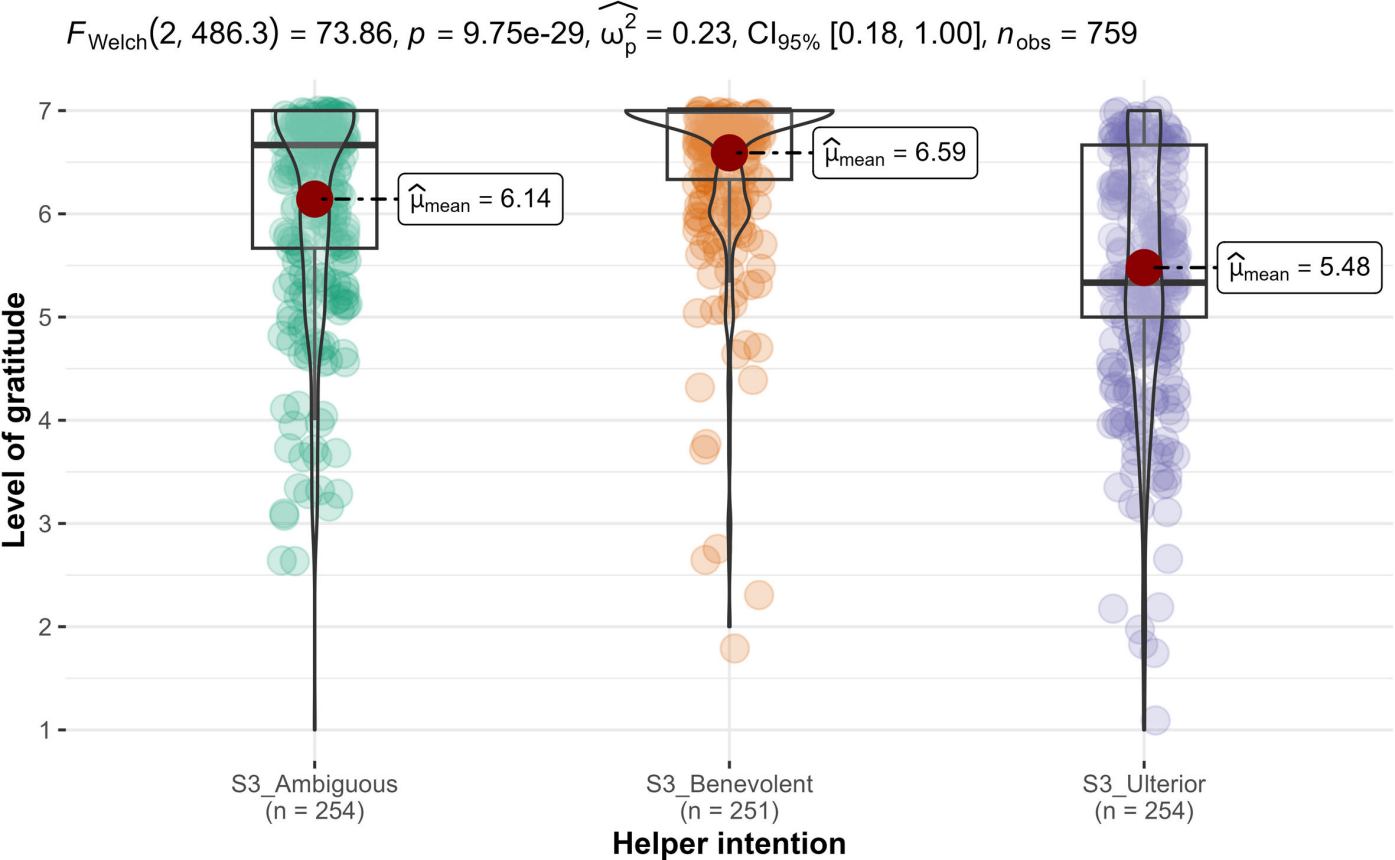
Study 3: helper intention impact on gratitude. Note: scale is from 1 to 7; higher values indicate stronger feelings of gratitude.

**Figure 7.**
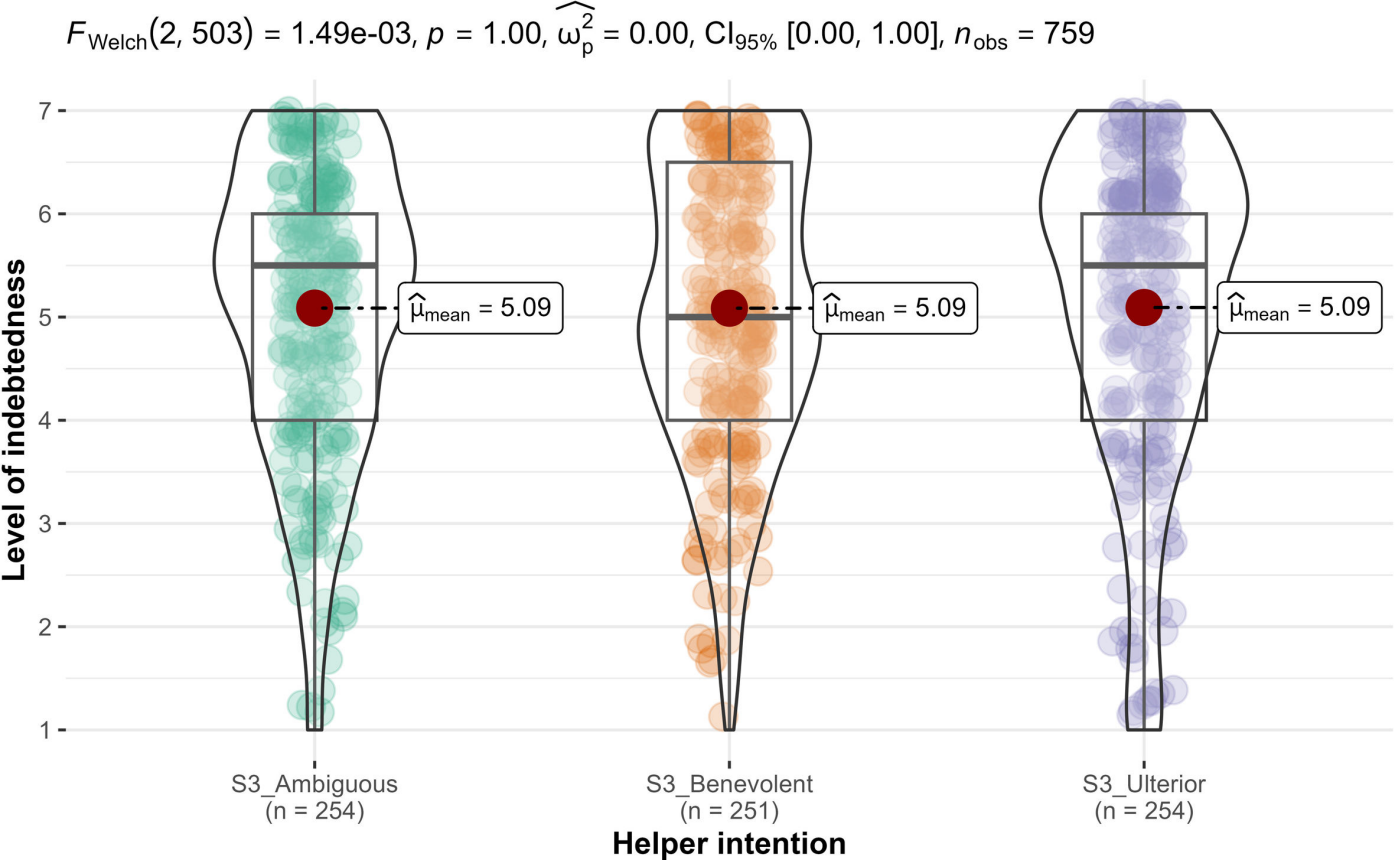
Study 3: helper intention impact on indebtedness. Note: scale is from 1 to 7; higher values indicate stronger feelings of indebtedness.

We conducted one-way ANOVAs and found support for helper intention’s impact on gratitude (*F*_2, 756_ = 70.9, *p* < 0.001; $\eta_{p}^{2}$* *= 0.16, 90% CI [0.12, 0.20]; figure 6), but not on indebtedness (*F*_2, 756_ = 0, *p* = 1.0; $\eta_{p}^{2}$* *= 0.00; figure 7). Therefore, we concluded support for hypothesis 7a, that gratitude is different across the three conditions (benevolent, selfish-ulterior and ambiguous), and—as expected—no support for hypothesis 8a (reframed from null hypothesis) that indebtedness is different across the three conditions.

Following the ANOVAs, we conducted *post hoc* contrasts analyses for hypotheses 7b and 7c and found support for feelings of gratitude in the ambiguous condition (*n* = 254; *M* = 6.14, s.d. = 1.09) as weaker than in the benevolent condition (*n* = 251; *M* = 6.59, s.d. = 0.79; *M_d_* = −0.45; *t*_756_ = −4.76, *p* < 0.001; *d* = −0.42, 95% CI [−0.60, −0.25]; H7b), but stronger than in the selfish-ulterior condition (H7c: *n* = 254; *M* = 5.48, s.d. = 1.24; *M_d_* = 0.66; *t*_756_ = 7.09, *p* < 0.001; *d* = 0.63, 95% CI [0.45, 0.81]; between benevolent and selfish-ulterior: *t*_756_ = 11.83, *p* < 0.001; *d* = 1.05, 95% CI [0.87, 1.24]).

Also, we conducted *post hoc* contrasts analyses for hypotheses 8b and 8c (reframed from null hypotheses) and—as expected—found no support for differences in indebtedness comparing the benevolent condition (*M* = 5.09, s.d. = 1.42) to both the ambiguous condition (*M* = 5.09, s.d. = 1.58; *M_d_* = 0; *t*_756_ = 0.01, *p* = 0.9948; *d* = 0.00, 95% CI [−0.17, 0.18]; H8b) and the selfish-ulterior condition (*M* = 5.09, s.d. = 1.43; *M_d_* = 0; *t*_756_ = −0.05, *p* = 0.969; *d* = 0.00, 95% CI [−0.18, 0.16]; H8c).

#### 3.1.2.5. Interaction between intent and emotions (gratitude versus indebtedness): extension analysis of a direct test to core hypothesis

**Figure 8.**
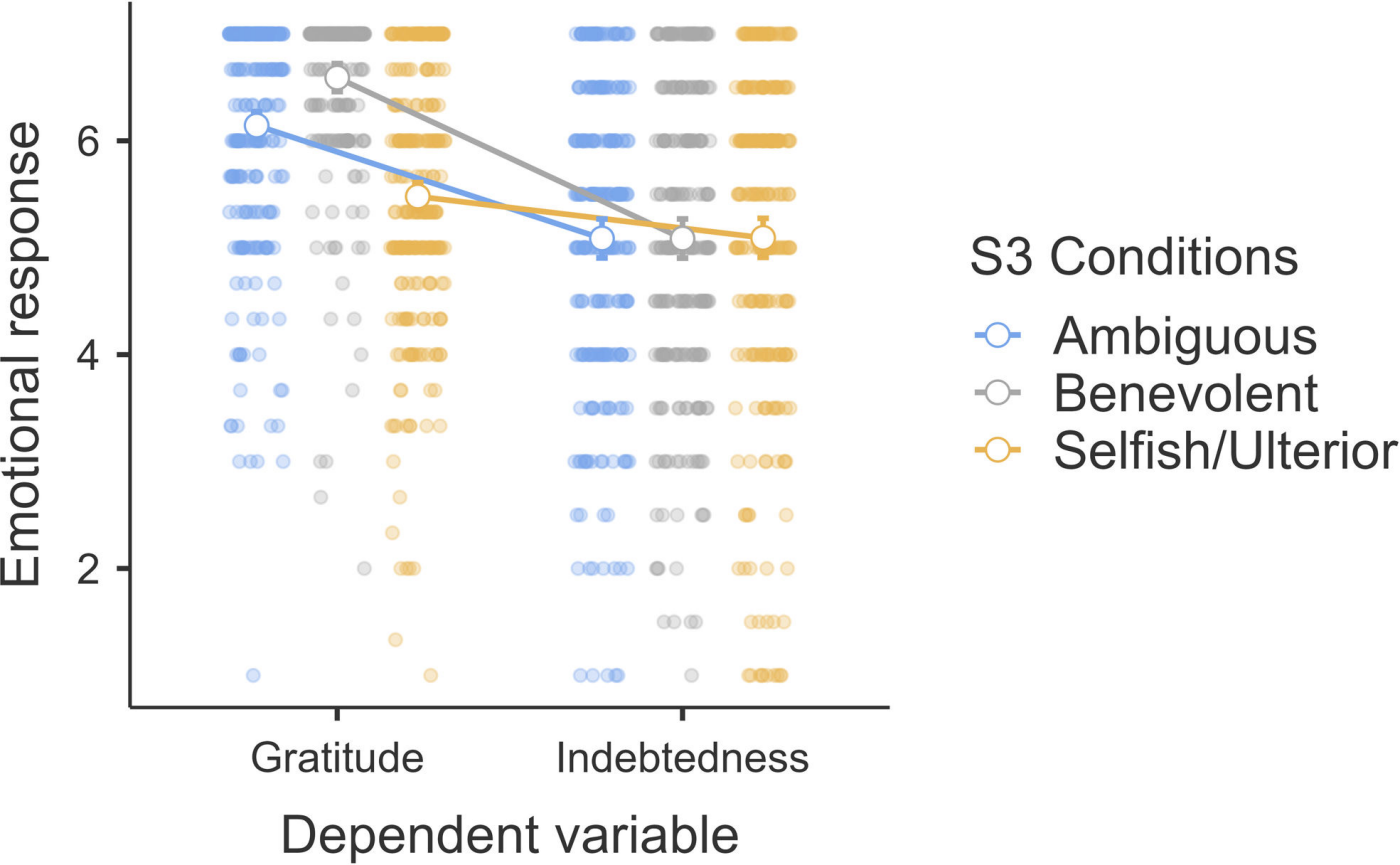
Study 3: interaction between helper intention and emotions (H7b/c + H8 b/c). Note: scale is from 1 to 7; higher values indicate stronger emotional response.

We conducted a mixed ANOVA examining the interaction between intent (benevolent versus selfish-ulterior versus ambiguous; between-subject) and emotion type (gratitude versus indebtedness; repeated) and found support for a main effect for emotion type (*F*_1, 756_ = 275.57, *p* < 0.001, $\eta_{p}^{2}$* *= 0.27, 95% CI [0.22, 0.31]), a main effect for intent (*F*_2, 756_ = 19.72, *p* < 0.001, $\eta_{p}^{2}$* *= 0.05, 95% CI [0.03, 0.08]) and an interaction between intent and emotion type (*F*_2, 756_ = 30.12, *p* < 0.001, $\eta_{p}^{2}$* *= 0.07, 95% CI [0.05, 0.10]; H7b/c and H8b/c combined; figure 8).

#### 3.1.2.6. Associations between helper intentions, gratitude and indebtedness

We conducted correlation tests (Pearson’s correlation) in the ambiguous condition and found support for more selfish intention as being negatively associated with gratitude (*r*_252_ = −0.50, 95% CI [−0.59, −0.40], *p* < 0.001), yet not associated with indebtedness (*r*_252_ = −0.01, 95% CI [−0.14, 0.11], *p* = 0.80).

### 3.2. Extensions: perceived expectations for reciprocity and reciprocity inclination

We added perceived expectations for reciprocity and reciprocity inclination as two extension dependent variables in study 3 and evaluated how they differ across conditions and are associated with gratitude and indebtedness.

**Figure 9.**
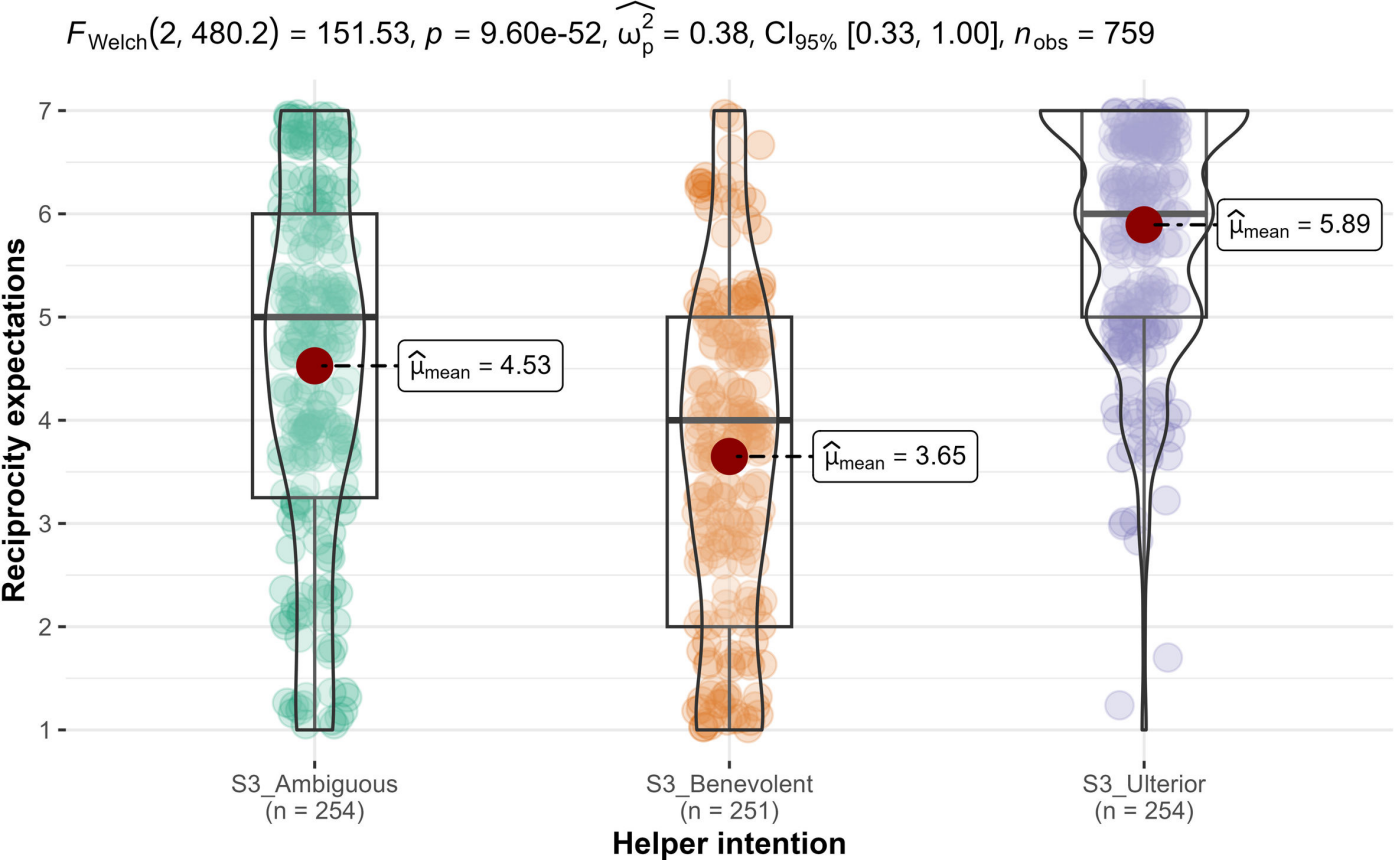
Study 3: helper intention impact on reciprocity expectations. Note: scale is from 1 to 7; higher values indicate higher reciprocity expectations.

First, we conducted independent samples *t*-tests (Welch’s; two-tailed) and found support for higher perceived *expectations for reciprocity* in the selfish-ulterior condition (*n* = 254; *M* = 5.89, s.d. = 1.18) than in the benevolent condition (*n* = 251; *M* = 3.65, s.d. = 1.79; *M_d_* = −2.24; *t*_433_ = −17, *p* < 0.001; *d* = −1.51, 95% CI [−1.71, −1.31]; figure 9). We found support for hypothesis 11a over 11b.

**Figure 10.**
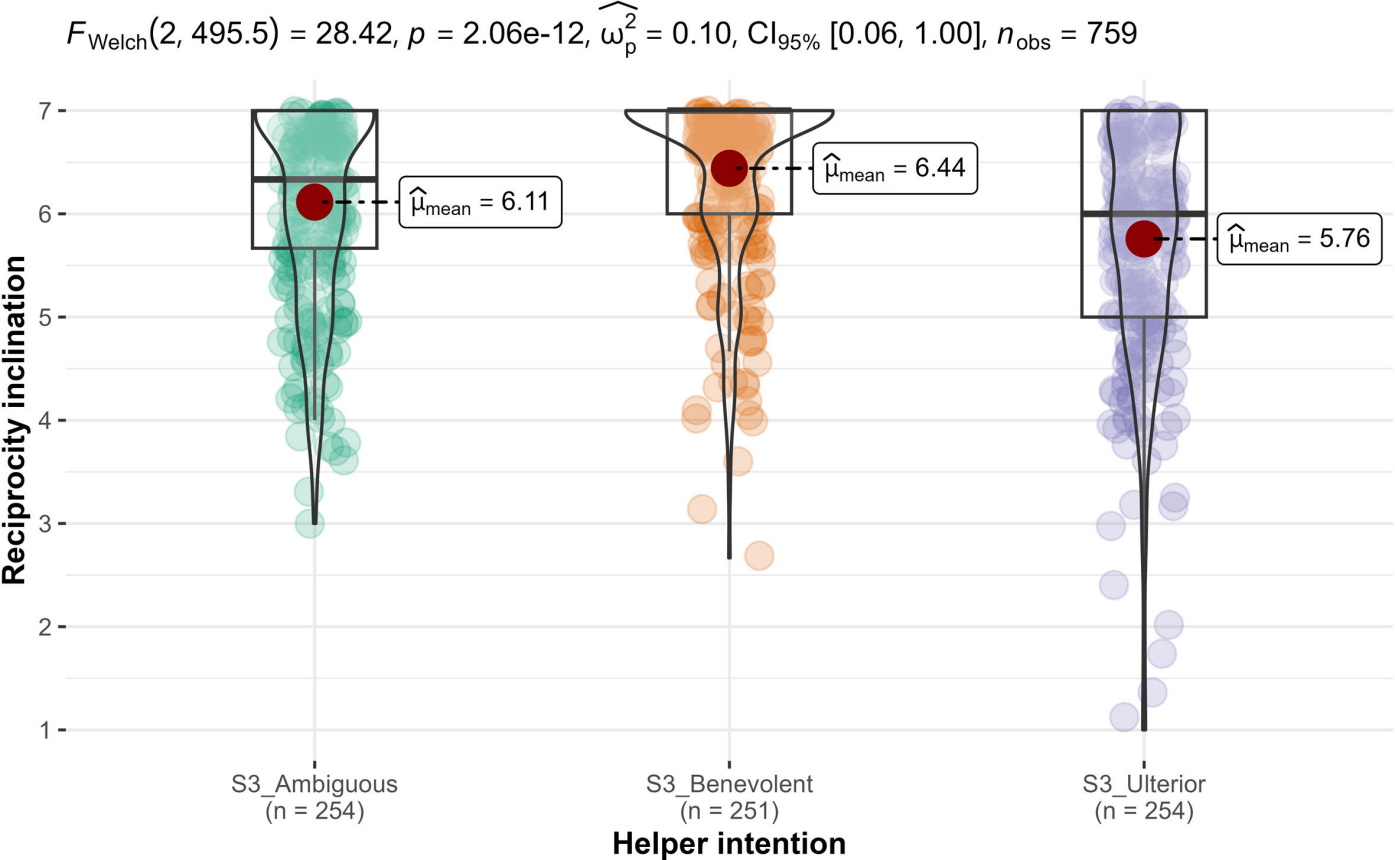
Study 3: helper intention impact on reciprocity inclination. Note: scale is from 1 to 7; higher values indicate higher reciprocity inclination.

Then, we conducted independent samples *t*-tests (Welch’s; two-tailed) and found support for higher *reciprocity inclination* in benevolent condition (*n* = 251; *M* = 6.44, s.d. = 0.84) than those in the selfish-ulterior condition (*n* = 254; *M* = 5.76, s.d. = 1.2; *M_d_* = 0.68; *t*_455_ = 7.4, *p* < 0.001; *d* = 0.66, 95% CI [0.48, 0.84]; figure 10). We found support for hypothesis 12b over 12a.

**Figure 11.**
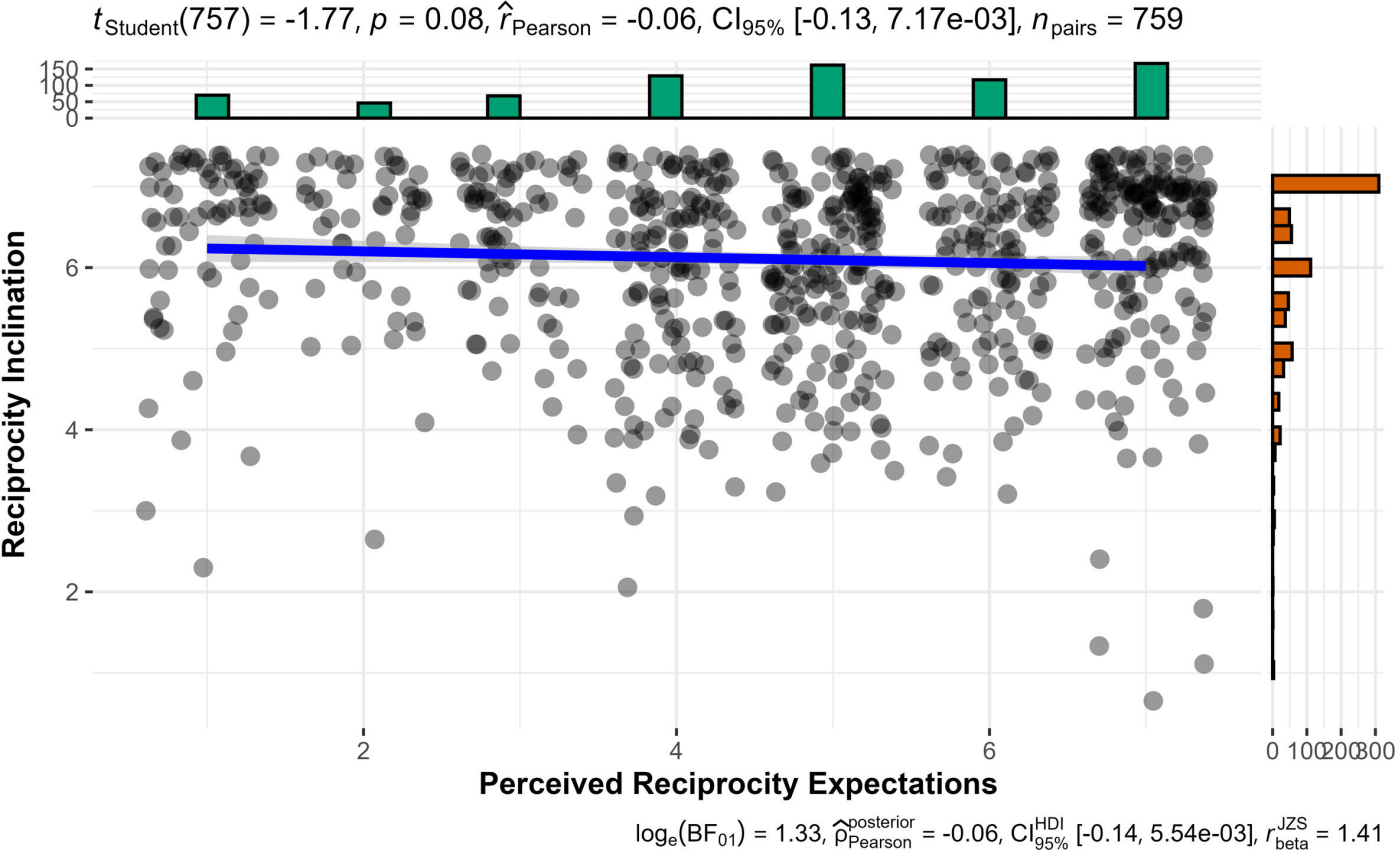
Study 3: the association between perceived expectations for reciprocity and reciprocity inclination.

We then conducted correlation tests (Pearson’s correlation) and found no support for a link between reciprocity inclination and perceived reciprocity expectations (*r*_757_= −0.06, 95% CI [−0.13, 0.01], *p* = 0.08; figure 11). We failed to find support for hypothesis 13 that perceived reciprocity expectations is correlated with reciprocity inclination.

We also conducted correlation tests (Pearson’s correlation) and found support for a negative correlation between perceived reciprocity expectations and gratitude (*r*_757_ = −0.28, 95% CI [−0.35, −0.22], *p* < 0.001) and a positive correlation between perceived reciprocity expectations and indebtedness (*r*_757_ = 0.17, 95% CI [0.10, 0.24], *p* < 0.001). We successfully found support for hypotheses 14 and 15 that gratitude and indebtedness are associated with perceived expectation for reciprocity.

Finally, we added exploratory correlations for the associations with reciprocity inclination. We found support for reciprocity inclination having a positive association with gratitude (*r*_757_ = 0.52 [0.46, 0.57], *p* < 0.001) and a weaker positive association with indebtedness (*r*_757_ = 0.29 [0.22, 0.35], *p* < 0.001; *z* for differences between correlations = 5.71, *p* < 0.001).

## 4. Discussion

In our replication and extensions registered report for the effects of helper intention on gratitude and indebtedness demonstrated by Tsang [1], results were mostly consistent with the findings reported in the target article (see table 8 for a summary).

### 4.1. Replication

Overall, we found that benevolent (versus selfish) intentions were more strongly associated with gratitude than with indebtedness. Comparison of hypotheses 2 and 3 in study 2 (H2: $\eta_{p}^{2}$ = 0.33 > H3: $\eta_{p}^{2}$ =0.001) as well as hypotheses 7b/c and 8b/c in study 3 (H7b/c: *d* = 0.42/0.63 > H8b/c: *d* = 0) both revealed that impact of helper intent on gratitude is much stronger than on indebtedness. These results are consistent with the conceptualization by Tsang [1] of gratitude and indebtedness as distinct emotions and with different emotional levels depending on perceived benefactors’ intentions.

Our replication reveals slightly weaker effect sizes for some of the effects compared to those reported in the original article. For example, the association between gratitude and indebtedness is weaker across conditions in study 2 (hypothesis 1a: original: *r* = 0.57; replication: *r* = 0.34) and in the ambiguous condition in study 3 (hypothesis 6: original: *r* = 0.42; replication: *r* = 0.23). However, all is still very consistent with the core hypotheses in the target article about the influence of helper intent on gratitude and indebtedness suggested by Tsang [1]. Overall, seven out of nine of the hypotheses in study 3 were consistent and shared remarkably similar effect sizes with the original article, indicating the robustness and replicability of the conceptualization by Tsang [1] on gratitude and indebtedness after more than one and a half decades.

Some may question the value of replication studies, asserting that highly cited studies are inherently reliable and replicable. We believe these reactions often stem from a hindsight bias (also known as the ‘knew-it-all-along phenomenon’) that many, including researchers, harbour towards replication studies. We previously demonstrated an ironic display of hindsight bias over the replicability of a classic experiment on hindsight bias (study 3; [46]). To try and address hindsight bias over the replicability of our target article, we conducted a prediction poll on Twitter/X on 26 March 2023 [47] and found that 24 out of 30 (80%) of the researchers in the community predicted an unsuccessful replication of studies 2 and 3 by Tsang [1], which is one of the lowest predictions among other targets included in the same Twitter/X poll. These predictions stand in strong contrast to the very successful replication we reported here and further highlights the importance of testing intuitions and the possible misperceptions that some may hold towards the importance of comprehensive independent registered reports of direct replication.

### 4.2. Extensions: perceived expectations for reciprocity and reciprocity inclination

We ran extensions examining the relationship between gratitude, indebtedness, perceived reciprocity expectations and reciprocity inclination. Our findings showed that: (i) perceived expectation for reciprocity was lower in benevolent helping intent than in selfish-ulterior helping intent; (ii) reciprocity inclination was stronger in receiving a benevolent helping than a selfish-ulterior helping; (iii) expectation for reciprocity was negatively associated with gratitude and positively associated with indebtedness; and (iv) there was no indication that expectations for reciprocity is associated with reciprocity inclination. Overall, we found empirical support for the hypothesis that gratitude and indebtedness are correlated to expectation for reciprocity, yet failed to find support for the association between expectation for reciprocity and reciprocity inclination.

Our findings support the argument by Watkins *et al*. [6] that expectation for reciprocity would be associated with higher indebtedness but lower gratitude. Provided that benevolent helping intent is associated with lower expectations for reciprocity, then according to the experimental paradigm of Watkins *et al*. [6], it would be associated with decreased indebtedness and increased gratitude. Our extensions help link between Tsang [1] and Watkins *et al*. [6] into a more comprehensive theory that higher benevolent intent is correlated with lower expectations and therefore higher gratitude than indebtedness.

### 4.3. Implications, limitations, and directions for future research

We concluded a successful replication, yet noted several limitations in the way we implemented the replication. First, we did not take into account the inflation from 2006 at which the study was first held, to 2023 at which our replication was held. The stimuli used in our $200 in 2006 is fairly equal to $300 in 2023. This was a dilemma that we acknowledged when we conducted our replication, but we decided not to change the stimuli. We considered the target’s claims and theory, but we did not find in the article or in any follow-up literature that indicated it as an important factor, and thus, we decided to conduct a direct replication for our current study without changing the value. Our successful replication with similar effect sizes with the target further supports the conclusion that this was not a critical factor.

Second, we have not performed a systematic review and meta-analysis of the literature pointing to the findings in the literature that built up on the target article. The scope for this direct replication with extensions was rather narrow and purely focused on the empirical effort to reproduce and replicate the original findings, and thus we decided to keep our literature review concise, mostly to explain how the target article was embedded in the broader literature. We believe our successful replication can serve as a cornerstone for future systematic reviews on research of gratitude and indebtedness and provide empirical evidence for a more comprehensive meta-analysis since the studies by Tsang [1] were first conducted.

Third, we added the expectation for reciprocity as an extra dependent variable on top of the original manipulation check of helper intentions, given that the scenario also explicitly mentioned expectations in the benevolent (versus selfish) condition. This could be a duplication. We decided to keep what we replicated and what we extended discrete for our direct replication, but we see room for regarding the expectation for reciprocity as a manipulation check, combining it with the original dependent variable of perceived helpers’ motivations.

Fourth, we deviated from the target article’s design by having participants take part in both the replication of study 2 and the replication of study 3 in random order. This may potentially introduce order effects, where responses to one scenario might influence responses to subsequent scenarios. However, by randomizing the sequence of two studies for each participant, we minimized potential biases. Our exploratory analyses indicated that this had little to no impact on the findings, with results consistent when comparing the full sample (as pre-registered) to studies presented first (exploratory). Given the strong alignment between the effects observed in the original research and our replication results, and the exploratory analyses, we believe order had little to no impact on our findings.

Our replication and extension takes the first step in combining the key relevant experimental paradigm of Tsang [1] and Watkins *et al*. [6] about the influence of helper intent and expectation for reciprocity on gratitude and indebtedness, with a conceptual replication of Bartlett & DeSteno [2] and Peng *et al*. [16] about the relationship between gratitude, indebtedness and reciprocity. The strong association between helper intentions and expectations for reciprocity in our findings may serve as the empirical foundation to develop a comprehensive framework for explaining the influence of helper intentions on gratitude and indebtedness. By contrast, the absence of an association between expectations for reciprocity and reciprocity inclination in our results may be seen as lending support for the findings by Peng *et al*. [16] over that of Bartlett & DeSteno [2].

Following a successful replication of Tsang [1] and our extension of studying reciprocity, we recommend more regular replications in the field, to state theoretical factors and predictions that might impact the effects and future replications and to examine moderators like the amount of money or degree of favour involved. Together with the empirical support for the impact of helper intentions on gratitude and indebtedness found in this replication, we believe it would be ideal to conduct a comprehensive systematic review, two decades after the research by Tsang [1] was first conducted.

Lastly, although we found an association between reciprocity expectations and gratitude and indebtedness in these studies, the causal effect between these variables remains unclear. Future research could consider trying to manipulate perceived expectations for reciprocity and reciprocity inclination, to further test the causal relationship between helper intent, reciprocity, gratitude and indebtedness.

## Data Availability

We provided all materials, data, and code on [48]. Supplementary material is available online [49].

## Footnotes

^1^
Stage 2 note: we implemented this scale with 1 = ‘slight urge’ given that this was the scale used in the target article. For future research, researchers might consider the option of offering participants the option of ‘no urge’, either as an added 0 value or to replace the lowest value of 1.
